# Preclinical development of a miR-132 inhibitor for heart failure treatment

**DOI:** 10.1038/s41467-020-14349-2

**Published:** 2020-01-31

**Authors:** Ariana Foinquinos, Sandor Batkai, Celina Genschel, Janika Viereck, Steffen Rump, Mariann Gyöngyösi, Denise Traxler, Martin Riesenhuber, Andreas Spannbauer, Dominika Lukovic, Natalie Weber, Katrin Zlabinger, Ena Hašimbegović, Johannes Winkler, Jan Fiedler, Seema Dangwal, Martin Fischer, Jeanne de la Roche, Daniel Wojciechowski, Theresia Kraft, Rita Garamvölgyi, Sonja Neitzel, Shambhabi Chatterjee, Xiaoke Yin, Christian Bär, Manuel Mayr, Ke Xiao, Thomas Thum

**Affiliations:** 1 Institute of Molecular and Translational Therapeutic Strategies (IMTTS), Hannover Medical School, Carl-Neuberg-Str. 1, 30625 Hannover, Germany; 2 CARDIOR Pharmaceuticals GmbH, Feodor-Lynen-Str. 15, 30625 Hannover, Germany; 3 Division of Cardiology, Medical University of Vienna, Waehringer Guertel 18-20, 1090 Vienna, Austria; 4 Institute of Molecular and Cell Physiology, Hannover Medical School, Carl-Neuberg-Str. 1, 30625 Hannover, Germany; 5 Institute for Neurophysiology, Hannover Medical School, Carl-Neuberg-Str. 1, 30625 Hannover, Germany; 6 Department of Diagnostic Imaging and Oncoradiology, University of Kaposvár, Guba S. Street 40, Kaposvár, 7400 Hungary; 7 Axolabs GmbH, Fritz-Hornschuch-Straße 9, 95326 Kulmbach, Germany; 8 The James Black Centre, King’s College, University of London, 125 Coldharbour Lane, London, SE5 9NU UK; 9 REBIRTH Center for Translational Regenerative Medicine, Hannover Medical School, Carl-Neuberg-Str. 1, 30625 Hannover, Germany

**Keywords:** Cardiac hypertrophy, Heart failure

## Abstract

Despite proven efficacy of pharmacotherapies targeting primarily global neurohormonal dysregulation, heart failure (HF) is a growing pandemic with increasing burden. Treatments mechanistically focusing at the cardiomyocyte level are lacking. MicroRNAs (miRNA) are transcriptional regulators and essential drivers of disease progression. We previously demonstrated that miR-132 is both necessary and sufficient to drive the pathological cardiomyocytes growth, a hallmark of adverse cardiac remodelling. Therefore, miR-132 may serve as a target for HF therapy. Here we report further mechanistic insight of the mode of action and translational evidence for an optimized, synthetic locked nucleic acid antisense oligonucleotide inhibitor (antimiR-132). We reveal the compound’s therapeutic efficacy in various models, including a clinically highly relevant pig model of HF. We demonstrate favourable pharmacokinetics, safety, tolerability, dose-dependent PK/PD relationships and high clinical potential for the antimiR-132 treatment scheme.

## Abstract (web summary)

miR-132 was shown to drive pathological cardiac remodeling, a hallmark of heart failure. Here, the authors show that an antisense inhibitor of miR-132 has favourable pharmacokinetics, safety-tolerability and preclinical efficacy in mouse and porcine models of heart failure.

## Introduction

Adverse structural remodeling of the left ventricle due to myocardial infarction (MI) is a common pathological feature leading to heart failure (HF)^1^. We previously demonstrated increased cardiomyocyte expression of the miR-212/132 family during pathological cardiac conditions^2^. Transgenic mice overexpressing the miR-212/132 cluster (miR-212/132-TG) develop pathological cardiac remodeling and die prematurely from progressive HF^2^. Using both knockout and antisense strategies, we have shown miR-132 to be both necessary and sufficient to drive the pathological growth of cardiomyocytes in a murine model of left ventricular pressure overload^2^. Based on the findings, we propose that miR-132 may serve as a therapeutic target in HF therapy.

Here we report mode of action details of a locked nucleic acid based antisense inhibitor of miR-132 (antimiR-132). We show translational evidence for therapeutic efficacy both in vitro and in vivo, in mice and in a clinically highly relevant pig model of HF. Finally, we demonstrate evidence for the antimiR-132 administration to be safe and well tolerated.

## Results and discussion

### Reversal of HF in mice by miR-132 antagonism

**Fig. 1:**
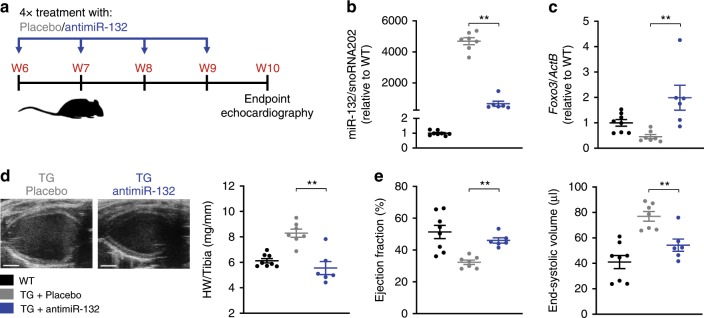
Therapeutic potential of antimiR-132 treatment. **a** Study outline of antimiR-132 application (20 mg/kg, i.p.) to miR-212/132 transgenic (TG) mice compared to placebo treatment (0.9% NaCl solution) and wildtype (WT) mice. **b** Functional miR-132 level. **c** MiR-132 target gene expression (Forkhead Box Protein O3, *Foxo3*). **d** Representative images of the parasternal long axis (PLAX) view of the left ventricle (LV) (scale bar = 2 mm) and heart weight (HW) vs. Tibia length ratio of mice. **e** Echocardiographic evaluation of ejection fraction and end-systolic volume. WT: *n* = 8, TG + NaCl: *n* = 7, TG + antimiR-132: *n* = 6. Data are mean ± s.e.m; **P < 0.01; unpaired two-sided Mann–Whitney *U* test.

To test the concept of miR-132 inhibition, we developed and optimized a synthetic, locked nucleic acid mixmer antisense oligonucleotide with fully phosphorylated backbone against miR-132 (antimiR-132). First, we tested this inhibitor in a target overexpression (miR-212/132- TG mice) model of HF. At the age of 6 weeks, miR-212/132-TG mice displayed severe left ventricular (LV) hypertrophy, reduced ejection fraction (EF) and cardiac dilatation. To test potential reverse remodeling effects, antimiR-132 (i.p., 20 mg/kg weekly) or placebo (0.9% NaCl solution) were given at this advanced HF stage for 4 consecutive weeks (Fig. 1a). AntimiR-132 treatment reduced functional miR-132 levels in cardiac tissue of miR-212/132-TG mice (Fig. 1b) and recovered expression levels of *Foxo3* (Forkhead Box Protein O3), a previously identified downstream target of miR-132^2^, showing successful target engagement (Fig. 1c). MiR-132 inhibition resulted in reduced cardiac mass, improved EF and reduced ventricular dilatation (Fig. 1d, e, Supplementary Table 1). Thus, pharmacological miR-132 inhibition improved cardiac function and reduced cardiac dilatation in the target-overexpression model of HF, highlighting the potential for clinical use in HF patients, for whose an increased cardiac expression level of the miR-212/132 cluster has been demonstrated^3^.

### AntimiR-132 ameliorates cardiomyocyte dysfunction

**Fig. 2:**
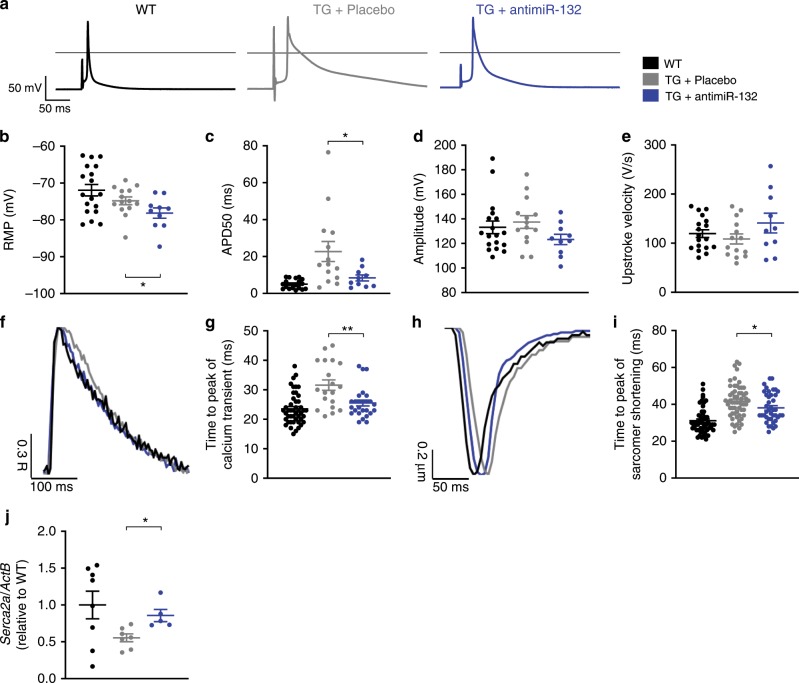
Functional properties of single cardiomyocytes. **a** Representative action potential traces of adult ventricular cardiomyocytes derived from wildtype (WT), miR-212/132 transgenic (TG) mice treated with placebo (0.9% NaCl solution) or antimiR-132. **b** Resting membrane potential (RMP). **c** Action potential duration at 50% level of repolarization (APD50). **d** Action potential amplitude. **e** Upstroke velocity. (WT: *n* = 18 cells, TG + NaCl: *n* = 14 cells, TG + antimiR-132: *n* = 10 cells) **f** Representative normalized calcium transients. **g**, Time-to-peak (ttp) of calcium transients at 3 Hz stimulation frequency. (WT: *n* = 46 cells, TG + NaCl: *n* = 19 cells/, TG + antimiR-132: *n* = 26 cells) **h** Representative normalized sarcomere shortening. **i** Time-to-peak (ttp) of sarcomeric contraction at 3 Hz stimulation frequency. (WT: *n* = 52 cells, TG + NaCl: *n* = 57 cells, TG + antimiR-132: *n* = 41 cells). **j** MiR-132 target gene expression in heart tissue (Sarcoplasmic/Endoplasmic Reticulum Ca^2+^ ATPase 2, *Serca2a2*). (WT: *n* = 8, TG + NaCl: *n* = 7, TG + antimiR-132: *n* = 5.) Data are mean ± s.e.m; *P < 0.05; **P < 0.01; unpaired two-sided Mann–Whitney *U* test.

To understand the favorable effect of miR-132 inhibition on cardiac muscle function (Fig. 1d, e) on top of previous studies^2^, we performed proteomic profiling of neonatal rat cardiomyocytes (NRCM) overexpressing miR-132 (Supplementary Fig. 1a). In total, 1783 proteins were detectable in at least three samples, from which 165 proteins showed significantly altered expression (Supplementary Fig. 1b). Using enrichment analysis, we found a majority of proteins that were regulated after miR-132 transfection to be related to contractile function (Supplementary Fig. 1c). We thus next compared the electrophysiological, calcium handling and sarcomere shortening parameters of singularized ventricular cardiomyocytes from adult wildtype (WT), untreated and antimiR-132-treated miR-212/132-TG mice. Representative traces of whole-cell patch clamp experiments in the current clamp mode are shown in Fig. 2a. Mean resting membrane potential (RMP) values of cardiomyocytes from the three different groups were comparable (Fig. 2b). However, action potentials (AP) of miR-212/132-TG mice were significantly prolonged as compared to WT cardiomyocytes (Fig. 2c). This was caused by slowing of the repolarization phase and expansion of the plateau phase, mimicking the shape of AP of failing human ventricular myocytes^4,5^. Treatment with antimiR-132 reconstituted AP durations to normal values (Fig. 2c). AP amplitudes and upstroke velocities were not different between the groups (Fig. 2d, e), suggesting sodium channels to be unaffected by miR-132. At all stimulation frequencies, miR-212/132-TG cardiomyocytes showed significantly slower times to peak (ttp) of calcium transients in comparison to WT cardiomyocytes (Fig. 2f, g; Supplementary Table 2). Treatment of TG cardiomyocytes with antimiR-132 significantly accelerated ttp of calcium transients (and different phases thereof) (Fig. 2g). Next, we measured sarcomere shortening of ventricular cardiomyocytes at increasing pacing frequencies from wildtype, untreated and antimiR-132-treated miR-212/132-TG mice, to validate the role of miR-132 in contractile function at the single cell level. MiR-132 overexpression led to significant prolongation of ttp of sarcomeric shortening at all stimulation frequencies in accordance to prolonged calcium transients (Fig. 2h, i; Supplementary Table 3). In line with the observations above, calcium handling and sarcomere shortening exhibited changes similar to that observed in isolated cardiomyocytes from human and animal models of HF^6–9^. Disorganization of the t-tubular network in HF and slowed down dyssynchronous activation of ryanodine receptors may account at least partially for the prolongation of intracellular calcium transients and contractions as well as a reduced *Serca2a* (Sarcoplasmic/Endoplasmic Reticulum Ca^2+^ ATPase 2) expression^10–12^. *Serca2a* is indeed a predicted target of miR-132^13^. To demonstrate direct proof of *Serca2a*’s mechanistic role in the mode of action of antmiR-132, we analyzed *Serca2a* expression in cardiac tissue and confirmed that *Serca2a* downregulation in miR-212/132 TG mice. In contrast, expression levels were restored in cardiac tissue form TG animals receiving antimiR-132 treatment (Fig. 2j).

Thus, elevation of miR-132 levels in cardiomyocytes has detrimental effects on contractile kinetics, which could be normalized by antimiR-132 treatment by, at least in part, restoring *Serca2a* expression. In addition to the known Foxo3-mediated antihypertrophic effects and normalization of autophagy, the miR-132-Serca2a axis is an additional mode of action for the cardiomyocyte specific effects of antimiR-132. This is supported by the fact, that cardiac miR-132 is mainly expressed in cardiomyocytes (Supplementary Fig. 2).

### Optimization of systemic antimiR-132 treatment

**Fig. 3:**
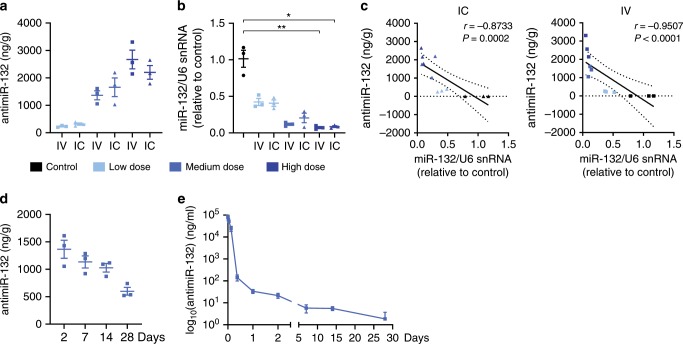
Pharmacokinetic properties of antimiR-132. **a** Cardiac tissue levels of antimiR-132 in healthy pigs 48 h post treatment for different dose levels (Control: None; Low = 1 mg/kg, Medium = 5 mg/kg and High = 10 mg/kg antimiR-132, *n* = 3 respectively) by intravenous (IV) or intracoronary (IC) application. **b** Functional cardiac tissue level of miR-132 in healthy pigs 48 h post treatment. **c** Correlation between antimiR-132 and miR-132 cardiac tissue levels in healthy pigs 48 h post treatment. **d** Time course of cardiac tissue levels of antimiR-132 in healthy pigs at different timepoints post treatment (5 mg/kg antimiR-132, IV, *n* = 3 respectively). **e** Plasma levels of circulating antimiR-132 in healthy pigs at different timepoints post treatment (5 mg/kg antimiR-132, IV, *n* = 3 respectively). Data are mean ± s.e.m; *P < 0.05, **P < 0.01; Kruskal–Wallis test with Dunn’s multiple comparison (Control vs. treatment groups) and linear regression using non‐parametric Spearman correlation.

To further assess the therapeutic potential of antimiR-132, we designed a large animal pharmacokinetic (PK) study to assess tissue exposure and distribution in the target tissue of our compound in pigs. Intravenous (IV) administration is a clinically preferred application route, however, many actual therapy approaches rely on alternative route of administrations (RoA), such as intracoronary (IC) perfusion, as often in the case for cardiac gene therapy studies. We found dose-dependent tissue exposure in the cardiac tissue samples comparable both for IV and IC administration of antimiR-132 (Fig. 3a, b). AntimiR-132 activity was confirmed by the reciprocal dose-dependent reduction of the target miR-132 level compared to untreated control animals. There was a strong inverse correlation between cardiac antimiR-132 concentration and functional miR-132 levels (Fig. 3c) independent of the RoA. The half-life of the compound in the cardiac tissue was calculated to be approximately 3 weeks (Fig. 3d) and for plasma a bi-phasic elimination of the compound with a rapid alpha phase and a long beta phase was found (Fig. 3e). The relatively long half-life in tissues and short bi-phasic half-life in plasma is well known in the oligonucleotide field^14,15^.

### AntimiR-132 treatment improves HF in a pig model

**Fig. 4:**
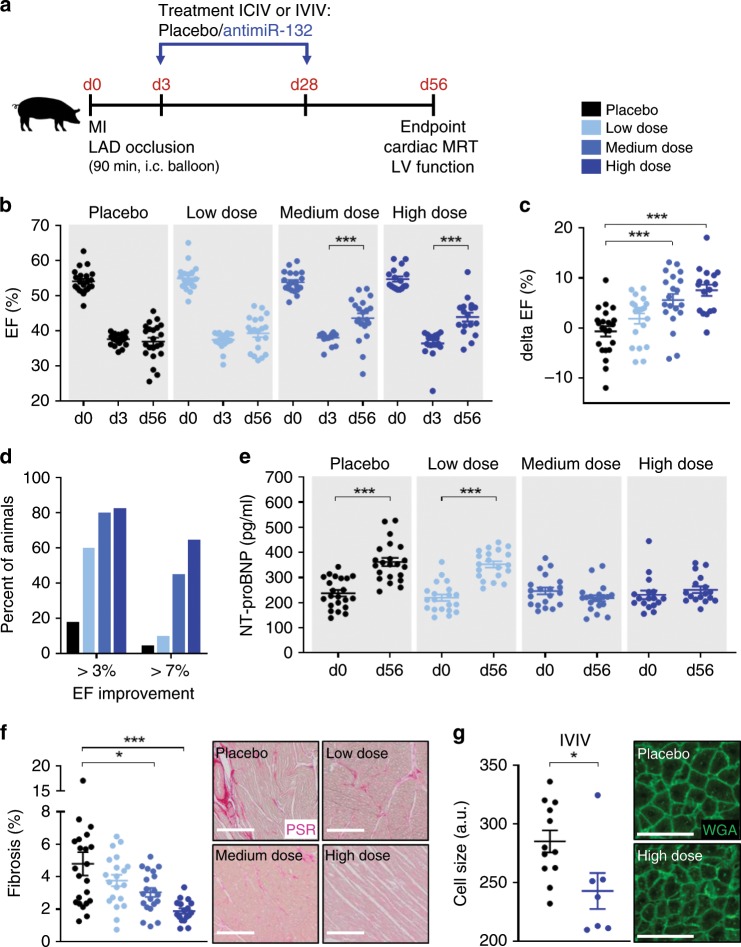
Proof-of-concept study in a large animal model of post-MI HF. **a** Study outline of treatment regimen (LAD = left anterior descending coronary artery). **b** Ejection fraction (EF) at baseline, day 3 and day 56 for different dosing groups of intracoronary/intravenous (ICIV) and intravenous/intravenous (IVIV) treated animals (Placebo: NaCl; Low = 1 mg/kg, Medium = 5 mg/kg and High = 10 mg/kg antimiR-132). **c** Functional improvement indicated by EF change from day 3 to day 56 (delta EF) for different dosing groups of ICIV and IVIV treated animals. **d** Responder analysis for different dosing groups of ICIV and IVIV treated animals. **e** N-terminal prohormone of brain natriuretic peptide (NT-proBNP) levels at baseline and day 56 for different dosing groups of ICIV and IVIV treated animals. **f** Quantification and representative micrographs of picrosirius red (PSR) staining of the left ventricular (LV) remote regions for different dosing groups of ICIV and IVIV treated animals (scale bar = 200 µm). **g** Quantification and representative micrographs of wheat germ agglutinin (WGA) staining for cardiac cell size measurement of the LV remote regions of IVIV treated placebo and high dose animals (scale bar = 50 µm). ICIV and IVIV: Placebo: *n* = 22, Low dose: *n* = 20, Medium dose: *n* = 20, High dose: *n* = 17. IVIV: Placebo *n* = 12, High dose: *n* = 7. Data are mean ± s.e.m; *P < 0.05, ***P < 0.001; unpaired two-sided Mann–Whitney *U* test (d3 vs. d56) or Kruskal–Wallis test with Dunn’s multiple comparison (Placebo vs. treatment groups).

On the basis of the PK data, we designed a pharmacodynamics (PD) study to test the antimiR-132 therapeutic efficacy in a large animal model of HF that closely mimics the clinical situation of post-MI patients who despite successful coronary reoxygenation therapy often develop HF. Our multi-arm study in a slow-growing (mangalica breed) pig model of post-MI HF allowed to evaluate various treatment schemes based on two different routes of administrations at different doses on cardiac function during a 56 days follow-up using serial cardiac magnetic resonance imaging (MRI) measurements (Fig. 4a). Placebo and 3 dose levels (Low, Medium and High corresponding to 1, 5, and 10 mg/kg, respectively) of antimiR-132 were applied either via IC infusion or IV injection at day 3. A second IV injection with the same dose was applied at day 28 in both groups (Fig. 4a). We opted for IV administration at this time point, due to clinical impracticality of IC at this later time point. In total, 135 pigs were subjected to an interventional MI procedure with 56 day follow-up (Supplementary Fig. 3a). Animal deaths during this study were related to periprocedural complications (12.6%) within 48 h post-MI prior to treatment initiation or due to fatal outcome of post-MI HF or iatrogenic cause. Notably, none of the deaths was associated with antimiR-132 treatment. Overall, 115 animals reached the day 56 endpoint, 36 of those were excluded due to limited development of LV dysfunction (EF ≥ 40% at day 3 post-MI based on MRI evaluation). Pigs were randomly assigned to the eight treatment groups on day 3. 79 animals were included (for final data analysis, defined as EF < 40% at day 3 post-MI) (Supplementary Fig. 3a). The degree of myocardial damage and the ensuing cardiac dysfunction may vary between animals despite standardized experimental MI procedure. In addition to cardiac imaging, plasma troponin T (TnT) measurements were used to assess myocardial damage, measured 3 days after MI, before start of treatment (Supplementary Fig. 3b). No differences were observed between the placebo and the various treatment groups, indicating no bias due to uneven distribution of study animals.

All animals developed subsequent characteristic signs of deteriorating LV function with a mean EF of 37.1% for the Placebo ICIV and 36.9% for the Placebo IVIV groups at endpoint (Supplementary Fig. 4a, b; Supplementary Tables 4, 5). AntimiR-132 dose-dependently improved cardiac function, reaching statistical significance for the medium and high ICIV groups (mean EF: 45.3% and 43.8%, respectively) and for the high dose IVIV group (mean EF: 44.1%) (Supplementary Fig. 4a, b; Supplementary Tables 4, 5). The functional improvement (delta EF = EF_day 56_ – EF_day 3_) significantly and dose dependently increased in all high dose groups and in the ICIV medium dose group (delta EF): 6.0% in the high ICIV group, 9.8% in the high IVIV group and 7.1% in the medium ICIV group, respectively (Supplementary Fig. 4c, e; Supplementary Tables 6, 7). These numbers correspond to a 6.6, 10.4 and 7.8% relative change to placebo animals (Supplementary Tables 8, 9). Based on the two RoA overlapping PK and target level activity profiles, we opted to combine the treatment arms and present the data in a pooled form. Overall, significantly increased EF at the study endpoint and functional improvement was found in the medium and high dose groups (mean EF: 43.6% and 44.0%, respectively, delta EF: 5.6% and 7.5%; respectively, delta EF relative to Placebo 6.3% and 8.2%, respectively (Fig. 4b, c; Supplementary Tables 10, 11, 12). Responder analysis, counting the percentage of animals in each group showing EF improvements (>3 or >7%), demonstrated a dose dependent therapeutic effect in both treatment arms (Supplementary Fig. 4d, f). Overall, of the combined groups 45.0% of the medium and 64.7% of the high dose group showed a delta EF of >7% on day 56 compared to 4.6% of placebo animals (Fig. 4d). As a sign of post-MI adverse remodeling, LV end-systolic volume (ESV) was significantly increased in the placebo group at endpoint. AntimiR-132 treatment in the medium and high dose groups effectively prevented this post-MI ESV enlargement (Supplementary Fig. 4g; Supplementary Table 10). This effect was even more visible by comparing change of LV volume data over time both as delta ESV (change between day 3 and day 56) (Supplementary Table 11) and normalized delta ESV to placebo (Supplementary Table 12). Testing for linear contrast revealed statistical significance, the change in delta ESV showed linear dose-dependent correlation (Supplementary Fig. 4h). Based on these data, we demonstrate a potent anti-remodeling effect of antimiR-132.

Additional data supporting the efficacy of our compound came from further biomarker studies and histological evaluations. N-terminal prohormone of brain natriuretic peptide (NT-proBNP) is a clinically highly relevant HF biomarker^16^. We measured NT-proBNP to validate the translational nature of the model to assess response to treatment by comparing levels at the study endpoint. We found that circulating NT-proBNP levels were significantly elevated in the placebo group at day 56 post-MI. In addition to the functional data mentioned above, the NT-proBNP elevation was significantly reversed in the medium and high dose groups (Fig. 4e). Histological assessment of fibrosis revealed a significant dose-dependent reduction of interstitial fibrosis suggesting improvements in the overall cardiac remodeling in the antimiR-132-treated groups (Fig. 4f). No inflammatory infiltration was observed in the cardiac tissue histology slides from any treatment groups. We also evaluated individual cardiomyocyte sizes in histological samples from the LV remote regions in our pig model. At the experimental endpoint, average size of the cardiomyocytes was significantly reduced in samples from the IVIV high dose (translationally most relevant group), compared to the placebo group (Fig. 4g). These findings are in line with the cardiomyocyte specific mode of action of our compound.

**Fig. 5:**
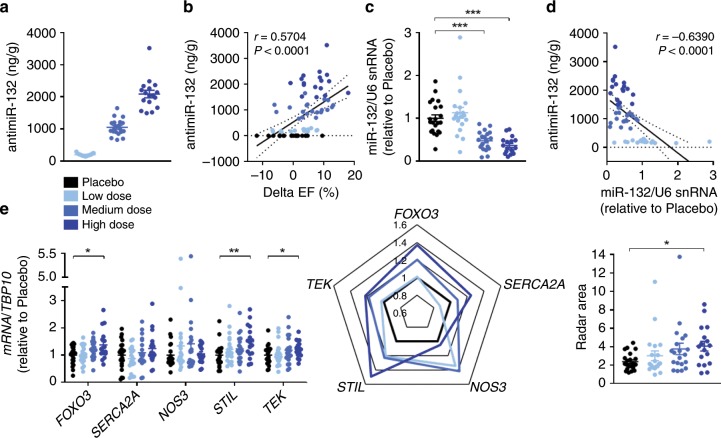
PK/PD relationship and target engagement panel. **a** Tissue levels of antimiR-132 detected in the left ventricular (LV) remote region for different dosing groups of intracoronary/intravenous (ICIV) and intravenous/intravenous (IVIV) treated animals (Placebo: NaCl; Low = 1 mg/kg, Medium = 5 mg/kg and High = 10 mg/kg antimiR-132). **b** Correlation between antimiR-132 tissue levels and functional improvement (delta ejection fraction (EF) = EF_day 56_ – EF_day 3_). **c** Functional tissue level of miR-132 detected in the LV remote region. **d** Correlation between antimiR-132 and miR-132 tissue levels. **e** Target de-repression in the LV remote region after antimiR-132 treatment (Forkhead Box Protein O3, *FOXO3*; Sarcoplasmic/Endoplasmic Reticulum Ca^2+^ ATPase 2, *SERCA2A*; Endothelial Nitric Oxide Synthase 3, *NOS3*; SCL/TAL1 Interrupting Locus, *STIL*; TEK Receptor Tyrosine Kinase, *TEK*). Radar chart depicting the target engagement panel. ICIV and IVIV: Placebo: *n* = 22, Low dose: *n* = 20, Medium dose: *n* = 20, High dose: *n* = 17. Data are mean ± s.e.m; *P < 0.05, **P < 0.01, ***P < 0.001; Kruskal–Wallis test with Dunn’s multiple comparison and linear regression using non‐parametric Spearman correlation.

By analysing drug concentrations in cardiac tissue samples at the study endpoint, we showed a dose-dependent linear increase of antimiR-132 (Fig. 5a) and a highly significant correlation with EF improvement proofing a strong PK/PD relationship (Fig. 5b; Supplementary Fig. 5a, c). A dose-dependent reduction of functional cardiac miR-132 levels was observed suggesting sufficient exposure by the administration and successful target engagement (Fig. 5c, d; Supplementary Fig. 5b, d).

### AntimiR-132 application is generally safe in all models

In the post-MI HF efficacy study, the pigs were carefully monitored for disease or drug related adverse effects by the veterinary team. No drug-related events or changes in mortality or morbidity were observed. There was no indication of drug-related local or systemic effects at any point. We also carefully monitored safety relevant hematology and laboratory chemistry parameters (including C-reactive protein, white blood cell counts, liver and kidney organ damage markers) during the pig study (baseline, day 3, 28 and 56 post-MI). There was no indication of drug-related effects at any point (Supplementary Fig. 6a). The expression of inflammatory mediators, such as *IL-6* (Interleukin-6) and *TNF-α* (Tumor necrosis factor alpha), was also not affected by treatments in cardiac tissue samples at the study endpoint (Supplementary Fig. 6b). Further, there was no difference in scar tissue size between the various treatment groups at the endpoint, indicating that antimiR-132 treatment does not affect the evolution of the fibrotic scar or the revascularisation in and around the infarcted area in the post-MI hearts (Supplementary Fig. 6c, d).

### Proof of target engagement for antimiR-132

To analyze the direct effects of antimiR-132 on miR-132-controlled gene networks in the target tissue, we established a target gene panel. We utilized RNA sequencing first to identify potential transcripts that interact with (target capture with biotinylated miR-132) and are regulated by miR-132 (target de-repression upon antimiR-132 treatment) in human induced pluripotent stem cell-derived cardiomyocytes (hiPS-CMs) (Supplementary Fig. 7a, b, c). Top regulated hits were derived by overlapping genes upregulated in both datasets and further analyzed by Gene Enrichment Analysis that unrevealed pathways highly associated with the cardiovascular system and disease (Supplementary Fig. 7d). This approach revealed several potential miR-132 target transcripts, such as *STIL* (SCL/TAL1 Interrupting Locus) and *TEK* (TEK Receptor Tyrosine Kinase), along with the cardiac relevant *NOS3* (Endothelial Nitric Oxide Synthase 3)^17^ (Supplementary Fig. 7e). This set of transcripts, combined with the known targets *FOXO3* as well as *SERCA2A*, was used as a target engagement panel. By this combined approach, we were able to demonstrate dose-dependent upregulation (i.e. de-repression) of targets in cardiac tissue from antimiR-132-treated pigs (Fig. 5e). When combined with functional miR-132 levels in these tissue samples, these data provide a multi-level proof of target engagement for antimiR-132 in vivo. In this proof-of-concept study we demonstrate that antimiR-132 effectively prevents maladaptive growth, remodeling and restore cardiac function via several cardiomyocyte specific pathways. We also demonstrate sufficient target tissue exposure, strong dose-dependent PK/PD relationship with our compound in a clinically relevant model. In addition, in vivo target engagement in cardiac tissue was demonstrated at microRNA and downstream target mRNA level. The dose-dependent improvement in cardiac function and the reversal of remodeling in both treatment arms reinforces the therapeutic potential of our compound.

We also demonstrate non-inferiority of IV vs. IC treatment based on the similar therapeutic effect and PK profiles. This enables us to utilize the IV administration scheme in subsequent clinical developments.

The compound was also safe and well tolerated at a wide pharmacological range.

Most importantly, we provide strong efficacy data and high clinical potential of our antimiR-132 treatment scheme in a large animal post-MI HF model, paving the way for clinical development of an innovative and effective therapy for patients with HF.

Indeed, the compound has recently entered the clinical stage with regulatory approval in June 2019 and subsequent starting of a Phase 1b clinical trial in HF patients (https://clinicaltrials.gov/ct2/show/NCT04045405).

## Methods

### Small animal studies and functional analysis

All mouse experiments were performed in accordance to the relevant regulations and guidelines of the Federation of European Laboratory Animal Science and with the approval of the governmental animal ethics committee LAVES (Nds. Landesamt für Verbraucherschutz und Lebensmittelsicherheit). For pharmacological studies, we used a previously generated^2^ transgenic mouse model overexpressing cardiomyocyte-specific miR-132 (C57BL/6N background). Transgenic animals were treated with antimiR-132 (LNA microRNA Inhibitor, Exiqon/Qiagen), as specified in the study design Fig. 1a. AntimiR-132 was diluted in 0.9% physiological saline that served as placebo as well. Mice received four intraperitoneal (i.p.) injections at a dose of 20 mg/kg/application. Wild type (WT) littermates served as controls. At the study endpoint, echocardiography was performed using Vevo 2100 (VisualSonics) under 2% isoflurane anesthesia, images recorded and analyzed using the Vevo LAB software 3.1.0 (VisualSonics).

### Isolation and culture of adult murine cardiomyocytes

Cardiac myocytes of adult mice were generated by a modified protocol of the retrograde perfusion method^18^. Briefly, mice received 0.1 ml heparin (2000 UI/kg body weight) under isoflurane anesthesia. After thoracotomy, the aorta was cannulated and the heart was perfused for 3 min with prewarmed Perfusion buffer (113 mM NaCl, 4.7 mM KCl, 0.6 mM KH_2_PO_4_, 0.6 mM Na_2_HPO_4_, 1.2 mM MgSO-7H_2_O, 0.032 mM phenol red, 12 mM NaHCO_3_, 10 mM KHCO_3_, 10 mM HEPES, 30 mM taurine, 0.1% glucose, 10 mM butanedione monoxime (BDM)). The buffer was switched to 1x Perfusion Buffer supplemented with 200 U/mL collagenase II (Worthington) and 12.5 µM CaCl_2_ and perfused for additional 10 min until the heart became soft. After removing the extraneous tissues including the atria, the digested heart was teased into small pieces and dissociated into single cell suspension. This digestion was stopped by addition of an equal amount of Stop buffer (perfusion buffer containing 10% FBS and 12.5 µM CaCl_2_) and the cell suspension filtered through a 100 µM cell strainer. Myocytes were separated from other cardiac cells by sedimentation and centrifugation. Calcium was gradually introduced before cells were plated on laminin-coated plates with plating medium (Medium 199, Hanks’ Balanced Salts (Thermo Fisher Scientific) pH 7.3 supplemented with 0.1232 g gluthation and 0.008 g BSA) and incubated at 37 °C and 5% CO_2_.

Cardiac fibroblasts were separated from myocyte and non-myocyte fractions by preplating at 37 °C and 1% CO_2_ for 45 min in minimal essential medium (Animed) supplemented with 4.2 mM NaHCO_3_, 2 ng/ml Vitamin B12, 1× Penicillin/Streptomycin and 10% FBS. Cardiac endothelial cells were prepared from the preplating supernatant using MACS MS Columns and CD146 (LSEC) MicroBeads for mouse (Miltenyi Biotec) according to the manufacturer’s instructions. Cardiac fractions for gene expression analyses were washed once with PBS and pelleted at 900 × *g* for 5 min before freezing in liquid nitrogen.

### Current clamp

Whole cell patch clamp analyses were performed in adult murine ventricular cardiomyocytes 6 h after isolation. Voltage changes were digitized with a Digidata 1550 and recorded with an Axopatch 200B amplifier (Molecular Devices). Pipettes were pulled from borosilicate glass (Harvard Apparatus) with resistances between 2 and 6 MΩ. The extracellular solution contained 140 mM NaCl, 5.4 mM KCl, 1.8 mM CaCl_2_, 1 mM MgCl_2_, 10 mM HEPES, 10 mM glucose, and pH 7.4 (adjusted with NaOH). The intracellular solution contained: 120 mM K-gluconate, 10 mM Na-Gluconate, 1 mM MgCl_2_, 3 mM Mg-ATP, 10 mM EGTA, 10 mM HEPES and pH 7.2 (adjusted with KOH). Agar bridges were used to connect the amplifier and the pipette solution. Results were corrected for junction potentials calculated using the JPCalc software^19^. Cells were hyperpolarized to −80 mV. AP were evoked by a current pulse of 1 ms duration. RMP or maximum diastolic potential (MDP), AP amplitude, AP duration at 50% repolarization (APD50) and upstroke velocity were determined. AP morphology was analyzed with the ClampFit software (Molecular Devices). Only cells with an input resistance of above 1 MΩ were used for analysis^20^.

### Single-cell sarcomere contraction and relaxation analysis

For single-cell sarcomere contraction and relaxation analysis^21–23^ a glass coverslip with single adherent cardiomyocytes was placed into a custom-made perfusion chamber. Myocytes were electrically stimulated with the MyoPacer EP Cell Stimulator (IonOptix) under constant perfusion with Hepes buffer (117 mM NaCl, 5.7 mM KCl, 1.2 mM NaH_2_PO_4_, 0.66 mM MgSO_4_, 10 mM glucose, 5 mM sodium pyruvate, 10 mM creatine, 20 mM HEPES, 1.0 mM EGTA, 1.25 mM CaCl_2_ adjusted to pH 7.4 and 37 °C)^24^. Only quiescent cardiomyocytes with clear striations and rod-like shape, which reacted to electrical pacing were randomly selected for single-cell experiments. For each cardiomyocyte, a rectangular region of interest (ROI) which typically included 15 to 20 sarcomeres, was adjusted and sarcomere length was calculated automatically by IonWizard software version 6.5 (IonOptix) using Fast Fourier Transformation analysis. Sarcomere contractions were recorded at 1 Hz, 3 Hz, and 5 Hz. Raw data was collected and stored with the IonWizard software version 6.5. For each cell at each stimulation frequency 20 to 30 single twitches were averaged. Parameters analyzed were diastolic sarcomere length (µm), contraction amplitude (measured as average shortening per sarcomere, µm), normalized maximum systolic shortening velocity in 1/s [(maximum slope in the contraction phase (-dL/dt), in µm/s) /contraction amplitude in s)], normalized maximum diastolic relaxation velocity in 1/s [(maximum slope of the relaxation phase (+dL/dt), in µm/s) /contraction amplitude in s], time to peak (ttp) and half relaxation time (hrt), both in s.

### Analysis of intracellular calcium transients

Intracellular Ca^2+^-transients of single cardiomyocytes were recorded using a dual excitation fluorescence photomultiplier system (IonOptix Corp.)^21–23^. Therefore, cardiomyocytes were loaded for 20 min at 37 °C and 5% CO_2_ with 1.5 μM fura-2 acetoxymethyl (AM) (Invitrogen) and washed twice for 15 min. The ROI was adjusted to the individual cardiomyocytes and fluorescence measurements were performed^21–23^. Ratio transients were recorded and stored using IonWizard software version 6.5 (IonOptix). Prior to analysis autofluorescence which was recorded separately from a group of not with fura-2 AM loaded cardiomyocytes using the same ROI as for loaded ones, was subtracted.

Parameters analyzed were diastolic ratio (R) and ratio amplitude (R), normalized maximum velocities of ratio increase in 1/s [maximum slope of calcium increase in(R/s)/ ratio amplitude in R] and ratio decay in 1/s [maximum slope of calcium decay in (R/s) /ratio amplitude in R] as well as time to peak and half decay time (RT50) of ratio transients, both in s.

### Isolation, culture, and treatment of NRCM

NRCM were isolated from newborn rats by enzymatic digestion^25^. NRCM were cultivated in minimal essential medium (Animed) containing 2 mg/L vitamin B12, 4.2 mM NaHCO_3_, 2 mM L-glutamine, 0.1 mM Bromdesoxyuridin (BrdU), 1% penicillin/streptomycin, and 5% FBS (Invitrogen) at 37 °C and 1% CO_2_. Cells were transiently transfected with pre-miRs using liposomal transfection. Briefly, premiR-132 (Ambion, PM10166) or premiR-control (Ambion, AM17111) and Lipofectamine 2000 (Thermo Fisher Scientific) were mixed with Opti-MEM I media (Thermo Fisher Scientific) and incubated separately for 5 min. Both solutions were combined, incubated for additional 20 min and added to the cells that have been washed with DPBS (Thermo Fisher Scientific). Cells were incubated for 4 h before the media were changed to fresh cultivation medium. Overexpression of miR-132 was monitored for 72 h after transfection.

### Protein isolation of the cellular membrane fraction

The Mem-PER Plus Membrane Protein Extraction Kit (Thermo Fisher Scientific) was used to isolate proteins from the cellular membrane based on a mild detergent-based and selective extraction protocol. In brief, around 5 × 10^6^ cells were scraped off the culture plate surface, centrifuged and washed. Cells were then permeabilized with 0.75 mL of Permeabilization Buffer, a mild detergent that allows the release of soluble cytosolic proteins. Then, 0.5 mL of Solubilization Buffer was added to solubilize membrane proteins. The fractions were stored at −80 °C. With this kit, membrane proteins with at least 1–2 transmembrane domains were extracted.

### Proteomics

Proteins of the cellular membrane fraction were digested with trypsin using an enzyme:protein ratio = 1:50 for in-solution digestion at 37 °C overnight. The digested samples were cleaned up by using C18 spin plate and separated by nanoflow HPLC (U3000 RSLCnano, EASY-SPRAY C18 column 75 µm × 50 cm) with a 2 h gradient and then analyzed with an Orbitrap Fusion Lumos (Thermo Fisher Scientific). Full MS was acquired on Orbitrap with a resolution of 120,000 and MS2 was acquired on linear ion trap using CID fragmentation for most abundant ions within 3 s cycle time and with dynamic exclusion enabled. Each sample was injected twice.

Proteome Discoverer 1.4.0.288 (Thermo Fisher Scientific) was used to search RAW files against UniProt/SwissProt Mammalia database (version 2015_02) using Mascot (version 2.3.1, Matrix Science). Search results were loaded into Scaffold (version 4.3.2, Proteome Science) for further validation with the following filters: peptide probability >95%, protein probability >99% with at least two unique peptides. Proteins were quantified using a Top3 precursor intensity method and missing values were imputed using a small number (1000). Proteins with a Student’s *t*-test *P* value < 0.05 were considered as significantly changed. Pathway enrichment analysis was performed using Enrichr^26^. All the data has been deposited in PRIDE with dataset identifier PXD015337 and 10.6019/PXD015337.

### PK studies and PoC study in a pig model of post-MI HF

The in-life phase of the studies was carried out in a collaboration with the Medical University Wien (MUW), Austria and the Institute of Diagnostic Imaging and Radiation Oncology (IDIRO) of the University of Kaposvar (now Medicopus Nonprofit Ltd.) in Kaposvar, Hungary. The study protocols have been reviewed and approved by the Animal Welfare Committee of the University of Kaposvar, Hungary for compliance with regulations prior to study initiation. All experiments were performed according to the Animal Study Registration Numbers SOI/31/26-11/2014 and SOI/31/01473-9/2017.

To assess pharmacokinetics (PK) in healthy pigs, animals were subjected to different doses (Low (1 mg/kg), Medium (5 mg/kg) or High (10 mg/kg)) of antimiR-132 via intravenous (IV) or intracoronary (IC) injections. A total volume of 20 mL solution/animal was applied at a flow rate of 80 mL/hour. Once completed, the tube was flushed with 5 mL sterile saline solution. Tissue samples were collected at 48 h post injection. Blood sampling was performed before (0 min) as well as at 3 min, 9 min, 30 min, 1 h, 3 h, 9 h, 24 h, and 48 h post injection. In the 5 mg/kg IV dose group additional tissue and blood samples were collected at 7, 14 and 28 days post injection. Untreated animals served as controls.

MI (PK study) and subsequent HF (multi-arm proof of concept (PoC) study) was induced by occlusion of the coronary artery for 90 min via a balloon catheter followed by removal of the balloon in 135 female, 4–5 months old Mangalica pigs (Hungarian breed of domestic pigs). The ensuing myocardial damage (ischemic/reperfusion injury) led to restricted myocardial function, progressive remodeling and signs of HF during the post-MI follow-up period.

Treatment groups of the multi-arm PoC study received a Low (1 mg/kg), Medium (5 mg/kg) or High (10 mg/kg) dose of antimR-132 applied either IC or IV on day 3 after MI and a second injection on day 28 after MI by IV application, as a short perfusion as described above. Control groups received saline solution, the vehicle of antimiR-132, as placebo. Prior to study start, the animals were assigned to either the IVIV or ICIV treatment arm and within these arms, animals were randomly assigned to the dosing groups. Further, animals were treated in a blinded fashion.

Blood sampling was performed at baseline, day 3 (pre-treatment), day 28 (pre-treatment) and day 56. On day 56 after MI, the animals were euthanized, and tissue samples were collected from pigs, frozen on dry ice, and stored at −80 °C for later processing.

### Functional parameter

Analysis of cardiac function was assessed by cardiac magnetic resonance imaging (cMRI) in a serial fashion, at baseline, day 3 (with late enhancement (LE)) and day 56 (with LE) post-MI. MRI assessment was performed at Medicopus using Siemens Magnetom Vision 1.5 Tesla field strength equipment (whole body magnetic resonance tomographic equipment; Siemens) under 3–5% isoflurane anesthesia. MRI images were evaluated by MUW using Segment for Windows software (version 1.9; Medviso AB). The primary endpoint was LV ejection fraction (EF) on day 56 post-MI. Animals showing an EF on day 3 of ≥40% were excluded from the study due to low response to the MI-model (see Supplementary Fig. 2a). Secondary endpoints were end-diastolic volume (EDV), end-systolic volume (ESV), stroke volume (SV), cardiac output (CO) and heart rate. Volumetric measurements of the left and right ventricle were performed using the freely available academic license software Segment for Windows software, and the global left and right ventricular EF and LV mass was calculated by measuring the EDV and ESV on short axis cine MRI images.

### Assessment of antimiR-132 tissue and plasma concentrations

AntimiR-132 levels from biological matrices were quantified at Axolabs using a HPLC assay that is based on the specific hybridization of the fully phosphorothioated LNA sequence with a complementary 16-mer PNA-probe labeled with an Atto425 fluorescence dye at the N-terminus. The PNA-probe with the sequence Atto425-OO-aac agt cta cag cca t was purchased from Panagene Inc.

Snap frozen tissue samples were sonicated in buffer containing proteinase K. In the resulting proteinase K lysates antimiR-132 was fully stable as all nuclease present in the biological matrix were degraded. For the subsequent hybridization step, an aliquot of the proteinase K lysates was mixed with a hybridization buffer containing 2.4 M urea in 12 mM Tris buffer pH 8 with 12% acetonitrile and the 16 mer PNA-probe.

The HPLC analysis was conducted on an Ultimate3000 HPLC system (Thermo Fisher Scientific) equipped with a low pressure gradient pump at a flow rate of 1 mL/min, a 96-well plate autosampler, a column compartment at 50 °C and a Shimadzu Florescence detector RF-20Axs (excitation wavelength: 436 nm; emission wavelength: 484 nm). HPLC buffer A was composed of 25 mM Tris buffer pH 8, 30% acetonitrile and 1 mM EDTA. HPLC buffer B contained 800 mM sodium perchlorate in HPLC buffer A. The HPLC column was washed after each sample using HPLC buffer C with 2 M sodium perchlorate, 3 M urea, 1 mM EDTA and 25 mM Tris buffer pH 8. Chromatography was performed on a DNA Pac PA100 column (4 × 250 mm, Thermo Fisher Scientific). Gradient elution of the compound was achieved by increasing the concentration of HPLC buffer B from 10% after 1 min to 45% at 9 min. An aliquot of 0.5 mg of tissue was injected per run in 100 µL of the final hybridized sample solution. The method was qualified for accuracy, precision, selectivity, carry-over, and linearity.

### Histology and fibrosis assessment

Heart tissue samples from the LV remote region were fixed in 4% formalin (Merck), embedded in paraffin and cut to 4–5 µm slices. For quantification of collagen and fibrosis, the samples were stained with Picro Sirius Red (PSR) and microscopy photographs were taken on an Olympus microscope IX83 (Olympus). Fibrosis was assessed based on collagen quantity by computerized planimetry using ImageJ (version 1.51) thresholding of four stained sections per animal, and mean values of the identical samples of the same animal were calculated.

Paraffin sections of the LV myocardium were visualized by wheat germ agglutinin (WGA) stain coupled to Alexa Flour 488 (Thermo Fisher Scientific). The cell surface area of cardiomyocytes was calculated using the NIS-Elements BR 3.2 package (Nikon Instruments Inc).

### Plasma sampling and laboratory diagnostics

Blood was collected into EDTA tubes for plasma sampling or coagulated for 30 min and centrifuged at 1300 xg for 10 min to generate serum samples. Hematology and biochemical analysis were performed by PraxisLab Kft (Budapest, Hungary) using fully automated instruments. Cell counts in anticoagulated whole blood sample and physical characteristics of the different cell types (red blood cells, white blood cells and platelets) were analyzed by a flow cytometry-based fully automated ADVIA 120 Hematology System (Siemens), using commercial reagents. Biochemical parameters were analyzed by spectrophotometric and potentiometric assay using the AU480 Chemistry Analyzer (Beckman Coulter) and commercial reagents. The results provide information on general health status and disease conditions, as well as model and potential treatment-related effects.

Troponin T (TnT) and N-terminal pro-brain natriuretic peptide (NT-proBNP) were assessed using an Enzyme-linked Immunosorbent Assay (ELISA) kit (Cloud Clone Corp) according to the manufacturer’s protocol. The enzyme-substrate reaction was measured spectrophotometrically with a Sunrise Multiplate reader (Tecan) at a wavelength of 450 nm ± 10 nm. The plasma concentrations of TnT and NT-proBNP were calculated based on the serial dilution of the protein standard.

### Differentiation and culture of hiPSC-derived cardiomyocytes

Human induced pluripotent stem cells (hiPSC)^27^ were maintained in feeder-free culture on Geltrex (Thermo Fisher Scientific) in StemMACS full medium with supplements (Miltenyi Biotec) using polystyrene plates (CELLSTAR; Greiner Bio-One).

Directed differentiation of hiPSCs towards cardiomyocytes was performed by modulating the Wnt pathway^28^. hiPSCs were split using Versene (Thermo Fisher Scientific) at 37 °C and then seeded onto a Geltrex-coated cell-culture dish in StemMACS supplemented with 2 μM Thiazovivin (Peprotech) (day −3) for 24 h. The medium was shifted to RPMI 1640 + GlutaMAX (Thermo Fisher Scientific) supplemented with albumin human recombinant (Sigma-Aldrich), L-Ascorbic acid 2-phosphate sesquimagnesium salt hydrate (Sigma-Aldrich) and the GSK3β inhibitor CHIR99021 (4 μM, synthesized by the Institute of Organic Chemistry, Leibniz University Hannover) when the cells reached a density of 70–80% (day 0). After 48 h, medium was changed to fresh media supplemented with 5 mM of the Wnt signaling inhibitor IWP2 (Peprotech). Following medium changes were performed every 48 h. From day 8 on, the cells were cultured in cardio culture medium containing RPMI 1640 + GlutaMAX supplemented with 1× B27 with insulin (Thermo Fisher Scientific), with a medium change every 2–3 days. CMs were purified using metabolic selection^29^ and studied on day 60 (calculated from day 0 of differentiation).

### Establishment of a target engagement panel

To identify transcripts that interact with and are possibly regulated by miR-132 RNA sequencing was performed in two sample sets generated to (a) capture direct interaction partners of miR-132 using a synthetic biotinylated hsa-miR-132-3p-duplex and to (b) to determine transcriptional changes after antimiR-132 treatment.

For the target capture experiment^30^ 10 nM of a synthetic biotinylated hsa-miR-132-3p-duplex (Integrated DNA Technologies) was introduced into human iPS-derived cardiomyocytes^27,28^ (hiPS-CM) by liposome-mediated transfection using Lipofectamine and Opti-MEM I (both from Thermo Fisher Scientific) according to the manufacturers protocol. Reference cells that underwent the same procedure were treated with a transfection mix where the synthetic miRNA was substituted by an equal volume of ultrapure RNase-free water. This transfection mix was replaced by culture medium (serum-free RPMI 1640 medium supplemented with Glutamax and B27, Thermo Fisher Scientific) after 12 h and cells lysed after an additional incubation for 24 h in culture medium. At endpoint, cell harvest was performed by scraping and centrifugation at 100 ×g for 10 min. Cells were lysed in Cell Lysis Buffer (10 mM Tris-Cl pH 7.5, 10 mM KCl, 1.5 mM MgCl_2_, 0.5% IGEPAL CA-630, 5 mM DTT, 60 U/mL RNaseOut, 1× cOmplete Mini EDTA-free protease inhibitor cocktail), frozen on dry ice for 5 min, thawed at room temperature, and precleared from debris by centrifugation at 16,200 ×g at 4 °C for 2 min. Then, NaCl was added to a final concentration of 1 M. The lysate was combined with Dynabeads MyOne Streptavidin C1 (Thermo Fisher Scientific) that have been blocked with Bead Blocking Solution (1 µg/µL BSA, 1 µg/µL Yeast tRNA in RNase-free water) at 4 °C overnight. After incubation with gentle agitation for 30 min, Dynabeads were washed three times with Wash Buffer (10 mM Tris-Cl pH 7.5, 10 mM KCl, 1.5 mM MgCl_2_, 0.5% IGEPAL CA-630, 1 M NaCl, 5 mM DTT, 60 U/mL RNaseOut, 1× cOmplete Mini EDTA-free protease inhibitor cocktail) to remove non-specifically bound molecules. Target transcripts bound to the Dynabeads via interactions to hsa-miR-132-3p-biotin were released by addition of 700 µL Qiazol Lysis Reagent (Qiagen) and total RNA further purified using the miRNeasy Micro Kit (Qiagen) according to the manufacturer’s instructions.

To determine the effect of miR-132 suppression on transcripts (target de-repression), hiPS-CM were treated with 100 nM antimiR-132 for 48 h. Cells treated with NaCl served as controls. At endpoint, RNA was isolated and sequenced along with samples from the target capture experiment. RNA sequencing of both experiments was performed by Biogazelle NV.

To identify target transcripts of miR-132 datasets from the capture as well as the de-repression experiment were compared. Since direct interactions between miR-132-3p and its targets are expected to be enriched in biotinylated miR-132 fraction and to be upregulated after antimiR-132 treatment, the analysis specifically focused on these datasets. Using the overlapping gene list, a pathway enrichment analysis was performed using Enrichr^26^. Genes involved in pathways relevant for cardiovascular disease were selected and expression levels of overlapping target hits were validated in hiPSC-CM treated with antimiR-132 as described above and along with known targets in heart tissue derived from the multi-arm PoC study in a pig model of post-MI HF. Results were represented as radar chart displaying multivariate data in the form of a two-dimensional chart of three or more quantitative variables represented on axes starting from the same point.

### Gene expression analysis

RNA of mouse heart tissue was isolated using the TriFast method (Peqlab) according to the manufacturer’s instructions, while porcine tissue biopsies of the LV remote region were isolated using miRNeasy Mini Kit (Qiagen). For mRNA detection, RNA samples were reverse transcribed with the iScript Select cDNA Synthesis Kit (BioRad; murine samples) or Verso cDNA Synthesis Kit (Thermo Fisher Scientific; porcine samples) and quantified on a Viia7 Real-Time PCR System using iQ SYBR Green supermix (BioRad) and gene-specific primer (murine *Foxo3*: 5′CAAAGCTGGGTACCAGGCTG3′, 5′TTCCACGGGTAAGGGCTTCA3′; murine reference gene *ActB*: 5′ATCAAGATCATTGCTCCTCCTG3′, 5′AGGGTGTAAAACGCAGCTCA 3′) or the ABsolute Blue QPCR Mix (Thermo Fisher Scientific) and TaqMan Gene Expression assays for human samples (Thermo Fisher Scientific; *Cacna1g*: Hs00367969_m1, *Bcl2L11*: Hs00367969_m1, *Nos3*: Hs01574665_m1, *Stil*: Hs00161700_m1, *Tek*: Hs00945150_m1, and *Tbp10*: Hs00427620_m1 as a reference gene) or pre-validated in pig tissue samples (Thermo Fisher Scientific; *Serca2a*: Ss03392433_m1, *Foxo3a*: Ss03374015_m1, *Nos3*: Ss03383840_u1, *Stil*: Hg05118054m1, *Tek*: Ss03388274m1, *Tnf-α*: Ss03391318_g1, *IL-6*: Ss03384604_u1, and *Tbp10*: Ss03391165_m1 as a reference gene) or for murine samples (Thermo Fisher Scientific; *Serca2a*: Mm01201431_m1, murine reference gene *ActB*: Mm02619580_g1).

To quantify miRNA levels, cDNA was generated using the TaqMan MicroRNA Reverse Transcription Kit (Thermo Fisher Scientific) and corresponding TaqMan MicroRNA RT Assays (Thermo Fisher Scientific). QRT-PCR was performed using the ABsolute Blue QPCR Mix (Thermo Fisher Scientific) and the TaqMan MicroRNA Assays (Thermo Fisher Scientific; miR-132-3p: 000457, snoRNA202: 001232 as a reference gene for murine samples and U6 snRNA: 001973 as a reference gene for porcine and human samples).

Data were collected and analyzed using the QuantStudio Real-Time PCR Software versions 1.1 and 1.3 (Thermo Fisher Scientific).

### Statistics

Data were analyzed using GraphPad Prism 7 software. Data are shown as mean ± s.e.m. *P* values were calculated using unpaired two-tailed unpaired Student’s *t-*test, two-sided Mann–Whitney *U* test (comparing two groups) or Kruskal–Wallis test with Dunn’s multiple comparison (comparing more than two groups) (as indicated). Linear regression analysis was based on non‐parametric Spearman correlation. Trend analysis was done by ANOVA, post-test for trend.

### Reporting summary

Further information on research design is available in the Nature Research Reporting Summary linked to this article.

## Supplementary information

Supplementary Information

Reporting Summary

## Data Availability

Proteomics data has been deposited in PRIDE with dataset identifier PXD015337 and 10.6019/PXD015337. The datasets generated during and/or analyzed during the current study are available from the corresponding author on reasonable request.

## Source data

Source data
