# Supplementary material for: Preclinical development of a miR-132 inhibitor for heart failure treatment
Source: Nat Commun. 2020 Jan 31;11:633. doi: 10.1038/s41467-020-14349-2 (PMC6994493; doi:10.1038/s41467-020-14349-2)
Supplement: Supplementary file 1 — Supplementary Information [file 41467_2020_14349_MOESM1_ESM.pdf]

Supplementary Information

Preclinical development of a miR-132 inhibitor for heart failure treatment;

by Ariana Foinquinos, Sandor Batkai, et al.

Supplementary Figure 1

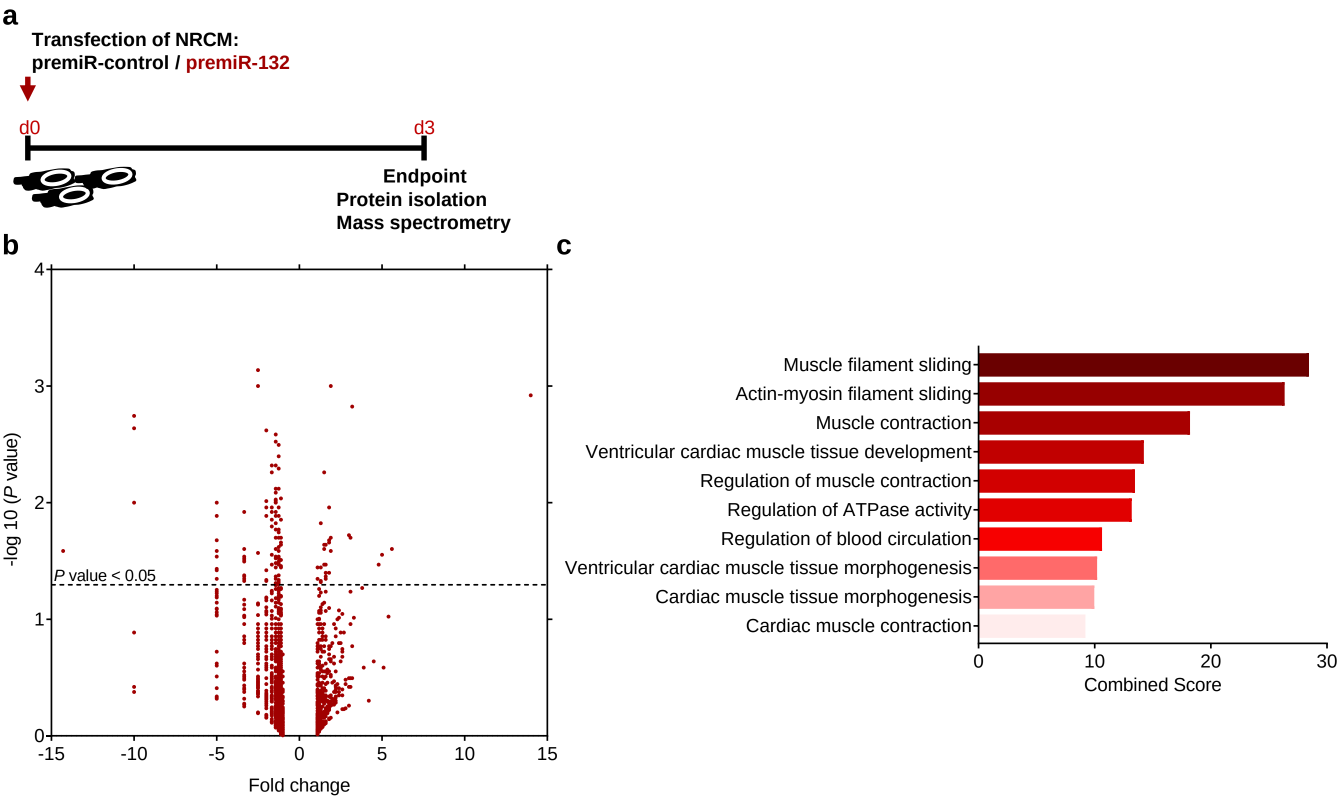

Supplementary Figure 1 Proteomic profiling after miR-132 overexpression. a, Study outline for the treatment of neonatal rat cardiomyocytes (NRCM) with premiR-132 and placebo (premiR-control) (100 nM). **b**, Volcano plot depicting dysregulated proteins. **c**, Bioinformatic protein enrichment analysis of the dysregulated proteins after premiR-132 treatment. *n*=3 per group. *P* values are generated by two-tailed unpaired Student's *t*-test.

Supplementary Figure 2

a

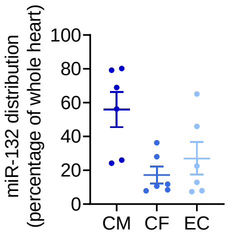

Supplementary Figure 2 Distribution of miR-132 expression among cardiac cell fractions. a, Distribution of miR-132 in cell fractions of murine heart: Cardiomyocytes (CM), cardiac fibroblasts (CF) and endothelial cells (EC). *n=6*. Data are mean ± s.e.m.

Supplementary Figure 3

a

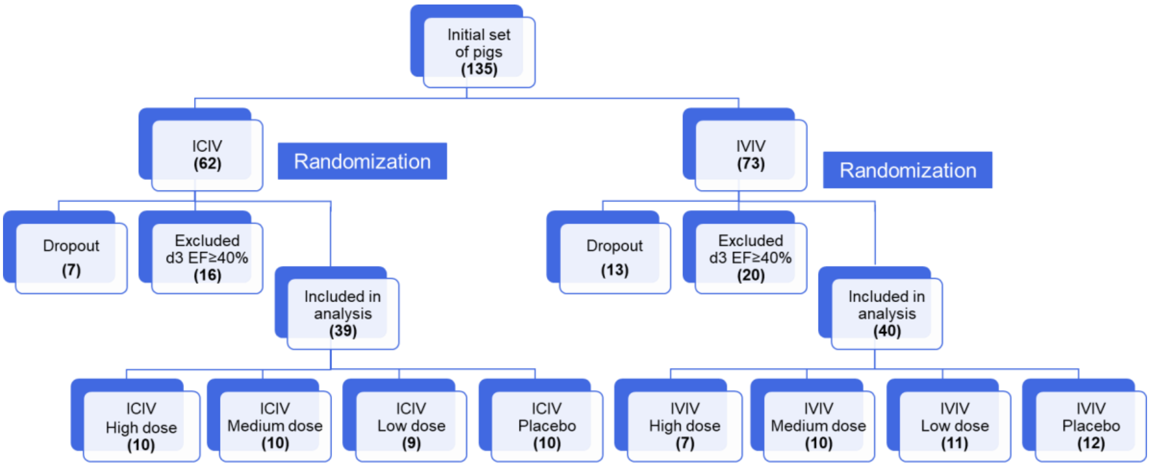

b

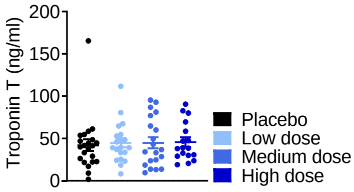

Supplementary Figure 3 Animal filtering strategy in proof-of-concept study in a large animal model of post-MI HF. a, 135 animals were assigned to either the intracoronary/intravenous (ICIV) or the intravenous/intravenous (IVIV) treatment arm and within these arms, animals were randomly assigned to the dosing groups (Placebo: NaCl, Low dose: 1 mg/kg, Medium dose: 5 mg/kg, High dose: 10 mg/kg). In total, 20 animals were considered as dropouts: 17 animals deceased within 48h post-myocardial infarction (MI) prior to the initiation of treatment, two animals deceased during the study due to non-drug-related issues. Overall, 115 animals reached the study endpoint at day 56. Due to biological (coronary anatomy) and technical variability, 36 surviving animals developed limited cardiac damage and post-MI ventricular dysfunction, defined as ejection fraction (EF) ≥ 40% at day 3 post-MI, and have been excluded. For the final data analysis of this study 79 animals were considered. The individual *n* number of each group is shown in this figure. **b**, Troponin-T levels at day 3 for different dosing groups of ICIV and IVIV treated animals. Placebo: *n*=22, Low dose: *n*=20, Medium dose: *n*=20, High dose: *n*=17. Data are mean ± s.e.m; Kruskal-Wallis test with Dunn's multiple comparison (Placebo vs. treatment groups).

Supplementary Figure 4

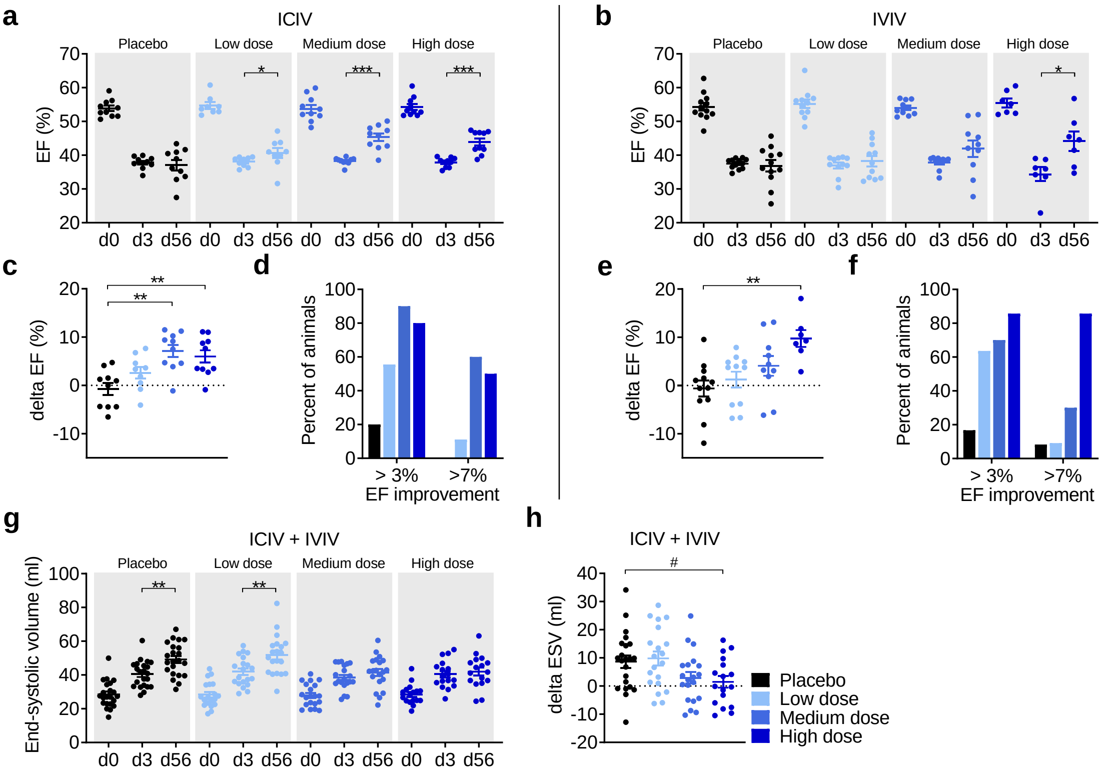

Supplementary Figure 4 Functional analysis in proof-of-concept study in a large animal model of post-MI HF. a, Ejection fraction (EF) at baseline, day 3 and day 56 for different dosing groups of intracoronary/intravenous (ICIV) treated animals. **b**, EF at baseline, day 3 and day 56 for different dosing groups of intravenous/ intravenous (IVIV) treated animals. **c**, Functional improvement indicated by EF change from day 3 to day 56 (delta EF) for different dosing groups of ICIV treated animals. **d**, Responder analysis for different dosing groups of ICIV treated animals. **e**, Functional improvement indicated by EF change from day 3 to day 56 (delta EF) for different dosing groups of IVIV treated animals. **f**, Responder analysis for different dosing groups of IVIV treated animals. **g**, End-systolic volume (ESV) at baseline, day 3 and day 56 for different dosing groups of both ICIV and IVIV treated animals. **h**, Functional improvement indicated by ESV change from day 3 to day 56 (delta ESV) for different dosing groups of both ICIV and IVIV treated animals. Linear trend was statistically significant. Placebo: NaCl; Low = 1 mg/kg, Medium = 5 mg/kg and High = 10 mg/kg antimiR-132. ICIV: Placebo: n=10, Low dose: n=9, Medium dose: n=10, High dose: n=10. IVIV: Placebo: n=12, Low dose: n=11, Medium dose: n=10, High dose: n=7. ICIV and IVIV: Placebo: n=22, Low dose: n=20, Medium dose: n=20, High dose: n=17. Data are mean ± s.e.m; $^{*}P < 0.05$, $^{**}P < 0.01$, $^{***}P < 0.001$; unpaired two-sided Mann-Whitney U test (d3 vs. d56) or Kruskal-Wallis test with Dunnett's multiple comparison (Placebo vs. treatment groups). $^{\#}P = 0.0058$; trend analysis using ANOVA, post-test for trend.

Supplementary Figure 5

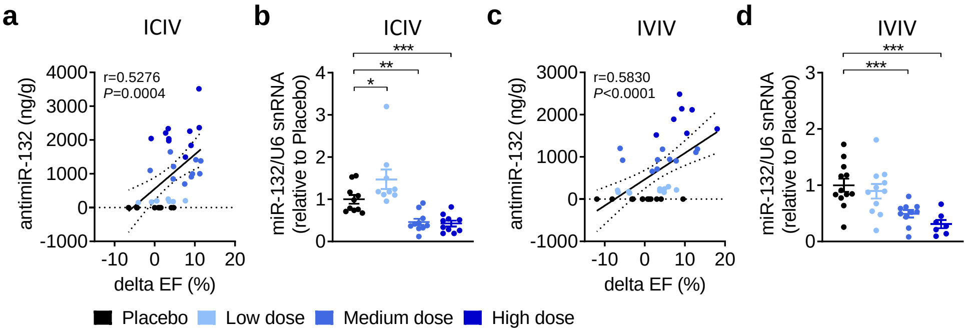

Supplementary Figure 5 PK/PD relationship in proof-of-concept study in a large animal model of post-MI HF. a, Correlation between antimiR-132 tissue levels in the left ventricular (LV) remote region and functional improvement of intracoronary/intravenous (ICIV) treated animals (delta ejection fraction (EF) = $EF_{day\ 56} - EF_{day\ 3}$). **b**, Functional tissue level of miR-132 detected in the LV remote region of ICIV treated animals. **c**, Correlation between antimiR-132 tissue levels in the LV remote region and functional improvement of intravenous/ intravenous (IVIV) treated animals (delta EF = $EF_{day\ 56} - EF_{day\ 3}$). **d**, Functional tissue level of miR-132 detected in the LV remote region of IVIV treated animals. Placebo: NaCl; Low = 1 mg/kg, Medium = 5 mg/kg and High = 10 mg/kg antimiR-132. ICIV: Placebo: n=10, Low dose: n=9, Medium dose: n=10, High dose: n=10. IVIV: Placebo: n=12, Low dose: n=11, Medium dose: n=10, High dose: n=7. Data are mean ± s.e.m; *$P < 0.05$; **$P < 0.01$; ***$P < 0.001$; Kruskal-Wallis test with Dunnett's multiple comparison and linear regression using non-parametric Spearman correlation.

Supplementary Figure 6

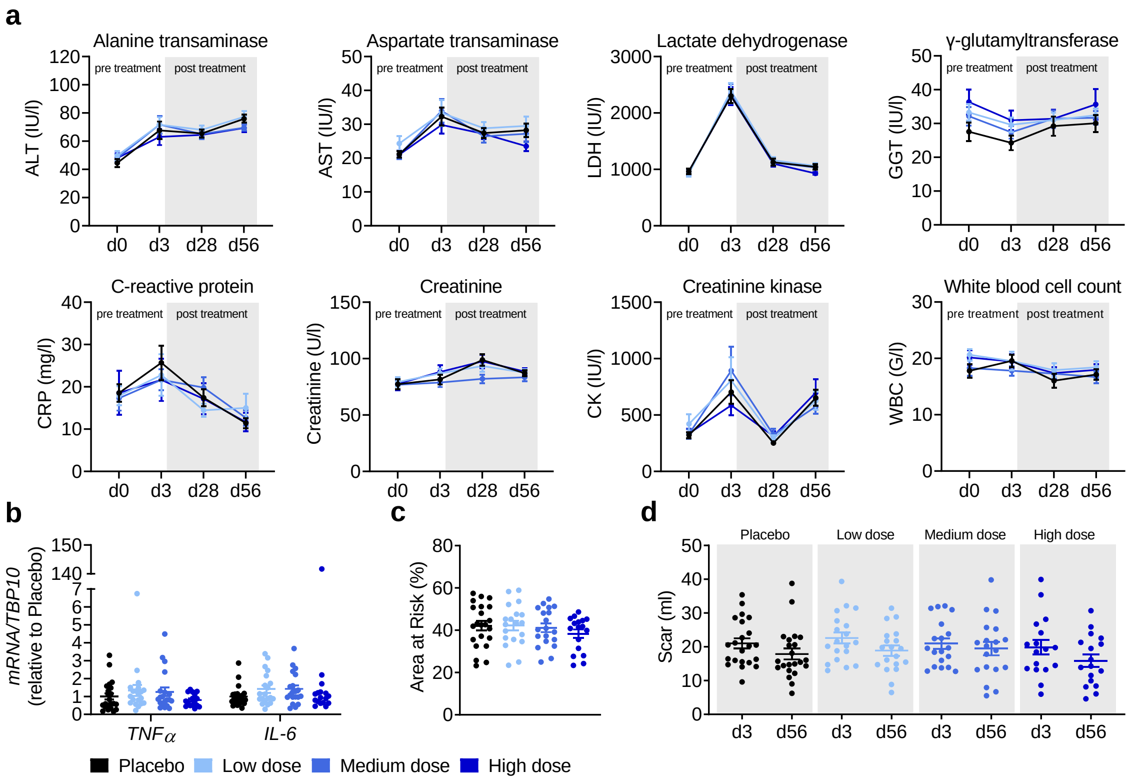

Supplementary Figure 6 Safety data of antimiR-132 in proof-of-concept study in a large animal model of post-MI HF. a, Lab chemistry parameters in plasma at baseline, day 3, day 28 and day 56 for different dosing groups of intracoronary/intravenous (ICIV) and intravenous/intravenous (IVIV) treated animals. **b**, Inflammatory marker gene expression (Tumor necrosis factor alpha, *TNFAα*, Interleukin-6, *IL-6*). **c**, Area at risk at day 3 for different dosing groups of ICIV and IVIV treated animals. **d**, Scar size at day 3 and day 56 for different dosing groups of ICIV and IVIV treated animals. Placebo: NaCl; Low = 1 mg/kg, Medium = 5 mg/kg and High = 10 mg/kg antimiR-132. ICIV and IVIV: Placebo: *n*=22, Low dose: *n*=20, Medium dose: *n*=20, High dose: *n*=17. Data are mean ± s.e.m. Kruskal-Wallis test with Dunn's multiple comparison.

Supplementary Figure 7

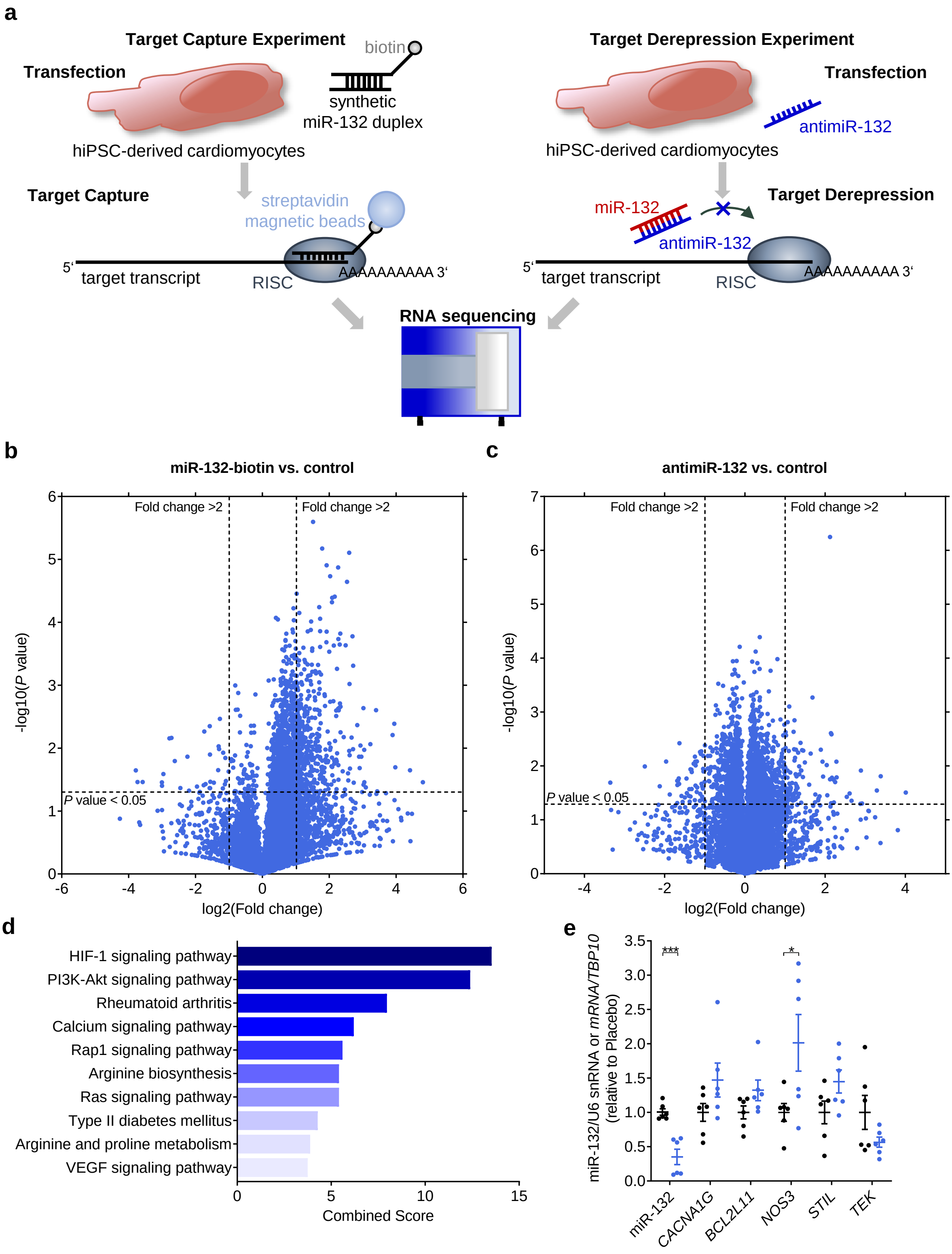

Supplementary Figure 7 Development of target engagement panel for antimiR-132. a, Study outline of miR-132 target capture using synthetic biotinylated miR-132-3p-duplex (10 nM transfected for 36h) and target de-repression after treatment with antimiR-132 (100 nM for 48h) performed in cardiomyocytes derived from human induced pluripotent stem cells (hiPSC). **b**, **c** Volcano plot depicting RNA sequencing results of differentially regulated transcripts after target capture or

de-repression compared to untreated control cells. *n*=3 per group. **d**, Gene enrichment analysis of overlapping genes upregulated in both datasets identified pathways highly associated with the cardiovascular system and disease. **e**, Potential target gene expression in antimiR-132 treated iPSC-cardiomyocytes (Calcium channel, voltage-dependent, T type, alpha 1G subunit, *CACNA1G*; Bcl-2-like protein 11, *BCL2L11*; Endothelial Nitric Oxide Synthase 3, *NOS3*; SCL/TAL1 Interrupting Locus, *STIL*; TEK Receptor Tyrosine Kinase, *TEK*). Data are mean ± s.e.m. *$P < 0.05$, ***$P < 0.001$; two-tailed unpaired Student's *t*-test.

Supplementary Table 1 Measurement of murine left ventricular function in a model of heart failure.

	WT	TG + Placebo	TG + antimiR-132
**LV ejection fraction (%)**	51.4 ± 4.2	32.3 ± 1.4	46 ± 1.7**
**Fractional shortening (%)**	26.4 ± 2.6	15.3 ± 0.7	22.9 ± 1**
**Cardiac output (µl/min)**	17.7 ± 1.2	15 ± 0.7	20.7 ± 0.7**
**Cardiac mass (mg)**	77.5 ± 4.3	117.6 ± 2.1	102.9 ± 5.6
**End-diastolic volume (µl)**	82.6 ± 5.1	113.3 ± 3.6	99.9 ± 6.4
**End-systolic volume (µl)**	41.1 ± 5.2	77 ± 3.8	54.3 ± 4.9**
**Stroke volume (µl)**	41.6 ± 3	36.3 ± 1	45.6 ± 1.9**
**End-diastolic diameter (mm)**	4.3 ± 0.1	4.9 ± 0.1	4.6 ± 0.1
**End-systolic diameter (mm)**	3.2 ± 0.2	4.2 ± 0.1	3.6 ± 0.1**
**Heart rate (bpm)**	428.1 ± 16.7	411.6 ± 15.9	454.7 ± 14.2

Data are mean ± s.e.m.
**$P < 0.01$ between TG + Placebo and TG + antimiR-132; unpaired two-sided Mann-Whitney *U* test.

Supplementary Table 2 Parameters of cardiomyocyte calcium transients at different stimulation frequencies.

	1 Hz			3 Hz		
	**WT**	**TG + Placebo**	**TG + antimiR-132**	**WT**	**TG + Placebo**	**TG + antimiR-132**
**Diastolic baseline ratio**	1.06 ± 0.02	0.93 ± 0.06	0.94 ± 0.04	1.18 ± 0.03	1.05 ± 0.06	1.02 ± 0.04
**Ratio amplitude**	0.34 ± 0.03	0.46 ± 0.12	0.45 ± 0.05	0.35 ± 0.03	0.52 ± 0.13	0.49 ± 0.04
**Time to peak (ms)**	23.7 ± 0.9	31.3 ± 2.3	26.8 ± 1.3	23.5 ± 0.8	31.6 ± 1.8	25.5 ± 1**
**10% Time to peak (ms)**	2 ± 0.1	2.6 ± 0.2	2.5 ± 0.3	2.4 ± 0.2	4.6 ± 0.6	3.5 ± 0.6
**50% Time to peak (ms)**	7.3 ± 0.2	9.5 ± 0.5	8.5 ± 0.5	7.6 ± 0.3	10.8 ± 0.6	8.9 ± 0.5**
**90% Time to peak (ms)**	14.8 ± 0.6	18.9 ± 1.3	15.8 ± 0.8	15.1 ± 0.8	20.4 ± 1.2	16.3 ± 0.8**
**Half decay time (ms)**	163.5 ± 7.5	158.1 ± 17.3	157.3 ± 7.6	86.9 ± 2.4	86.8 ± 5.1	80.7 ± 1.9
**Ratio increase rate (1/s)**	86.4 ± 2.8	65.1 ± 3.7	81.1 ± 3.8*	92.9 ± 2	79 ± 4.1	88 ± 2.4
**Number of animals/cells**	6/60	3/18	3/31	6/46	3/19	3/26

Data are mean ± s.e.m.

**P* < 0.05, ***P* < 0.01 between TG + Placebo and TG + antimiR-132; unpaired two-sided Mann-Whitney *U* test.

Supplementary Table 3 Cardiomyocyte contraction and relaxation parameters at different stimulation frequencies.

	1 Hz			3 Hz			5 Hz		
	**WT**	**TG + Placebo**	**TG + antimiR-132**	**WT**	**TG + Placebo**	**TG + antimiR-132**	**WT**	**TG + Placebo**	**TG + antimiR-132**
**Diastolic sarcomere length (µm)**	1.79 ± 0.01	1.76 ± 0.01	1.78 ± 0.01*	1.79 ± 0.01	1.76 ± 0.01	1.78 ± 0.01	1.79 ± 0.01	1.76 ± 0.01	1.77 ± 0.01
**Contraction amplitude (µm)**	0.07 ± 0.01	0.08 ± 0.01	0.08 ± 0.01	0.04 ± 0	0.06 ± 0.01	0.05 ± 0.01	0.04 ± 0.01	0.05 ± 0.01	0.05 ± 0.01
**Time to peak (ms)**	37.8 ± 1.1	45.6 ± 1.4	43.7 ± 1.7	31.1 ± 1	41.7 ± 1.2	38.1 ± 1.2*	29.5 ± 0.8	39.9 ± 1.1	36.6 ± 1.2
**10% Time to peak (ms)**	7.1 ± 0.3	10.6 ± 0.5	8.7 ± 0.7**	7 ± 0.4	10.7 ± 0.4	8.3 ± 0.4***	6.6 ± 0.3	11.8 ± 0.5	8.7 ± 0.5***
**50% Time to peak (ms)**	18.8 ± 0.6	23.3 ± 0.7	21.1 ± 0.9	15.3 ± 0.5	21.6 ± 0.6	18.7 ± 0.7**	14.9 ± 0.5	21.5 ± 0.6	18.4 ± 0.7***
**90% Time to peak (ms)**	30.5 ± 0.9	36.5 ± 1.1	35 ± 1.6	24.3 ± 0.8	32.6 ± 0.9	29.5 ± 1*	23.1 ± 0.7	31.6 ± 0.8	28.7 ± 0.9*
**Half relaxation time (ms)**	24.1 ± 0.7	26.8 ± 1.2	25.9 ± 1.2	22.4 ± 0.8	24.7 ± 1.2	25.2 ± 1.3	20.5 ± 0.7	22.6 ± 1	23.3 ± 1.3
**Contraction rate (1/s)**	42 ± 1.2	37.7 ± 1.2	39.7 ± 1.6	54.9 ± 1.8	45.3 ± 1.3	46.4 ± 1.5	58 ± 1.3	46.3 ± 1.4	49.4 ± 1.6
**Number of animals/cells**	5/57	4/62	3/38	5/52	4/57	3/42	5/54	4/55	3/39

Data are mean ± s.e.m.

**P* < 0.05, ***P* < 0.01, ****P* < 0.001 between TG + Placebo and TG + antimiR-132; unpaired two-sided Mann-Whitney *U* test.

Supplementary Table 4 Serial measurement of left ventricular (LV) function by cMRI of animals treated ICIV in a pig model post-MI HF.

	Placebo			Low dose			Medium dose			High dose		
	**Baseline**	**Day 3**	**Day 56**	**Baseline**	**Day 3**	**Day 56**	**Baseline**	**Day 3**	**Day 56**	**Baseline**	**Day 3**	**Day 56**
**LV ejection fraction (%)**	53.9 ± 0.8	37.8 ± 0.6	37.1 ± 1.6	54.7 ± 1	38 ± 0.5	40.7 ± 1.4*	53.7 ± 1.2	38.2 ± 0.3	45.3 ± 1.1**	54.2 ± 0.9	37.9 ± 0.4	43.8 ± 1.1***
**LV mass (g)**	58.6 ± 3.3	61.4 ± 3.6	64.1 ± 8.9	52.8 ± 4.4	61.6 ± 4.4	76.6 ± 7.2	53.3 ± 3.6	62.2 ± 4.2	72.4 ± 4.6	57.5 ± 4.5	60.1 ± 3.5	66.2 ± 6.7
**End-diastolic volume (ml)**	68.2 ± 5.2	68.9 ± 3.7	82.1 ± 3.7*	69.9 ± 5	73.2 ± 3.8	84.5 ± 4	68.5 ± 4	68.7 ± 2.8	80.1 ± 1.8**	67.2 ± 3.3	67.8 ± 3.8	78.6 ± 3.8
**LVEDV Index (ml/kg)**	2.4 ± 0.2	2.5 ± 0.1	2.8 ± 0.2	2.5 ± 0.1	2.6 ± 0.1	2.7 ± 0.1	2.4 ± 0.2	2.4 ± 0.1	2.4 ± 0.1	2.5 ± 0.1	2.5 ± 0.2	2.5 ± 0.1
**End-systolic volume (ml)**	31.7 ± 2.7	42.9 ± 2.4	51.6 ± 2.3*	31.9 ± 2.7	45.3 ± 2.3	50.3 ± 3.2	31.8 ± 2	42.4 ± 1.7	43.8 ± 1.4	30.9 ± 1.8	42.2 ± 2.5	44.3 ± 2.6
**LVESV Index (ml/kg)**	1.1 ± 0.1	1.5 ± 0.1	1.7 ± 0.1	1.1 ± 0.1	1.6 ± 0.1	1.6 ± 0.1	1.1 ± 0.1	1.5 ± 0.1	1.3 ± 0.1	1.1 ± 0.1	1.6 ± 0.1	1.4 ± 0.1
**Stroke volume (ml)**	36.5 ± 2.6	26 ± 1.3	30.6 ± 2.1	38 ± 2.4	28 ± 1.5	34.2 ± 1.7*	36.8 ± 2.2	26.3 ± 1.1	36.3 ± 1.1***	36.3 ± 1.7	25.6 ± 1.4	34.3 ± 1.6***
**Cardiac output (l/min)**	3.5 ± 0.3	2.4 ± 0.3	2.2 ± 0.2	3.9 ± 0.3	2.2 ± 0.1	2.6 ± 0.1	3.5 ± 0.3	2.2 ± 0.2	2.9 ± 0.1**	3.4 ± 0.2	2.4 ± 0.2	2.6 ± 0.2
**Heart Rate (bpm)**	97.2 ± 6.4	89 ± 6.9	71.2 ± 4.4	99.7 ± 4.6	80.7 ± 4.9	77.8 ± 5.5	94.4 ± 3.2	86.3 ± 3.7	79.3 ± 2.4	95.5 ± 3.5	92.7 ± 5	76.5 ± 5.4*

Data are mean ± s.e.m.

*$P < 0.05$, **$P < 0.01$, ***$P < 0.001$ between Day 56 and Day 3 per dosing group; unpaired two-sided Mann-Whitney *U* test.

Supplementary Table 5 Serial measurement of left ventricular (LV) function by cMRI of animals treated IVIV in a pig model post-MI HF.

	Placebo			Low dose			Medium dose			High dose		
	**Baseline**	**Day 3**	**Day 56**	**Baseline**	**Day 3**	**Day 56**	**Baseline**	**Day 3**	**Day 56**	**Baseline**	**Day 3**	**Day 56**
**LV ejection fraction (%)**	54.3 ± 1.1	37.5 ± 0.4	36.9 ± 1.6	55.2 ± 1.3	37 ± 0.9	38.2 ± 1.6	54 ± 0.7	37.9 ± 0.6	41.9 ± 2.5	55.5 ± 1.3	34.4 ± 2.1	44.1 ± 2.9*
**LV mass (g)**	47.9 ± 3	55.7 ± 2.3	60.9 ± 3.3	48.2 ± 2.6	55.8 ± 3.3	62.3 ± 3.2	44.8 ± 2	50.3 ± 2.2	59.8 ± 3.5**	41.9 ± 2.4	51.9 ± 5.2	57.9 ± 3.8
**End-diastolic volume (ml)**	56.4 ± 3.6	61.5 ± 3.5	74.3 ± 3.8*	57.3 ± 4	62.1 ± 4.1	85 ± 5.4**	52.3 ± 3.1	55.9 ± 3.5	67.5 ± 6.8	56 ± 3	57.7 ± 3.5	68.2 ± 5.1
**LVEDV Index (ml/kg)**	2.2 ± 0.2	2.4 ± 0.1	2.6 ± 0.1	2.1 ± 0.1	2.3 ± 0.1	2.6 ± 0.1	2.1 ± 0.1	2.2 ± 0.1	2.3 ± 0.2	2.3 ± 0.2	2.5 ± 0.2	2.5 ± 0.2
**End-systolic volume (ml)**	25.8 ± 1.8	38.5 ± 2.2	47.2 ± 3.3	25.7 ± 2	39.2 ± 2.7	52.9 ± 4.1**	24.2 ± 1.6	34.6 ± 2.1	39.1 ± 4	25 ± 1.6	38.1 ± 3.2	38.6 ± 4.3
**LVESV Index (ml/kg)**	1 ± 0.1	1.5 ± 0.1	1.6 ± 0.1	0.9 ± 0.1	1.5 ± 0.1	1.6 ± 0.1	1 ± 0.1	1.4 ± 0.1	1.4 ± 0.1	1 ± 0.1	1.6 ± 0.2	1.4 ± 0.2
**Stroke volume (ml)**	30.6 ± 1.9	23 ± 1.3	27 ± 1.4*	31.7 ± 2.4	22.9 ± 1.5	32.1 ± 1.9**	28.2 ± 1.5	21.2 ± 1.5	28.4 ± 3.3	31 ± 1.7	19.6 ± 1.3	29.6 ± 2**
**Cardiac output (l/min)**	3.3 ± 0.3	2.1 ± 0.1	2.1 ± 0.2	2.9 ± 0.2	2.1 ± 0.2	2.9 ± 0.3*	2.6 ± 0.1	1.9 ± 0.1	2.4 ± 0.3	3.1 ± 0.3	1.4 ± 0.2	2.2 ± 0.2*
**Heart Rate (bpm)**	107.6 ± 6.6	94.4 ± 4.5	79.1 ± 5.6	94.5 ± 7.3	91.6 ± 7.2	90.9 ± 10.7	94.7 ± 7.3	91.7 ± 4.3	82.3 ± 4.3	98.8 ± 8.6	67.8 ± 6.9	74.8 ± 5.5

Data are mean ± s.e.m.

*$P < 0.05$, **$P < 0.01$ between Day 56 and Day 3 per dosing group; unpaired two-sided Mann-Whitney *U* test.

Supplementary Table 6 Changes in left ventricular (LV) function between day 3 and day 56. (ICIV treated animals) in a pig model post-MI HF.

	Placebo	Low dose	Medium dose	High dose
**delta LV ejection fraction (%)**	-0.7 ± 1.2	2.6 ± 1.2	7.1 ± 1.3**	6 ± 1.3*
**delta End-diastolic volume (ml)**	13.2 ± 4.8	11.3 ± 4.4	11.5 ± 3.6	10.8 ± 4.8
**delta End-systolic volume (ml)**	8.7 ± 3.1	5 ± 3.5	1.4 ± 2.7	2.1 ± 3.1

Data are mean ± s.e.m.

*$P < 0.05$, **$P < 0.01$; Kruskal-Wallis test with Dunn's multiple comparison.

Supplementary Table 7 Changes in left ventricular (LV) function between day 3 and day 56. (IVIV treated animals) in a pig model post-MI HF.

	Placebo	Low dose	Medium dose	High dose
**delta LV ejection fraction (%)**	-0.6 ± 1.6	1.2 ± 1.6	4.1 ± 2.1	9.8 ± 1.8**
**delta End-diastolic volume (ml)**	12.8 ± 3.5	23 ± 4	11.6 ± 5	10.6 ± 3.8
**delta End-systolic volume (ml)**	8.7 ± 3.3	13.7 ± 3	4.5 ± 3.1	0.5 ± 2.4

Data are mean ± s.e.m.

**$P < 0.01$; Kruskal-Wallis test with Dunn's multiple comparison.

Supplementary Table 8 Changes in left ventricular (LV) function between day 3 and day 56 relative to Placebo. (ICIV treated animals) in a pig model post-MI HF.

	**Relative to Placebo**		
	**Low dose**	**Medium dose**	**High dose**
**delta LV ejection fraction (%)**	3.3 ± 1.2	7.8 ± 1.3	6.6 ± 1.3
**delta End-diastolic volume (ml)**	-1.7 ± 4.4	-1.5 ± 3.6	-2.2 ± 4.8
**delta End-systolic volume (ml)**	-3.7 ± 3.5	-7.3 ± 2.7	-6.6 ± 3.1

Data are mean ± s.e.m.

Supplementary Table 9 Changes in left ventricular (LV) function between day 3 and day 56 relative to Placebo. (IVIV treated animals) in a pig model post-MI HF.

	**Relative to Placebo**		
	**Low dose**	**Medium dose**	**High dose**
**delta LV ejection fraction (%)**	1.9 ± 1.6	4.7 ± 2.1	10.4 ± 1.8
**delta End-diastolic volume (ml)**	10 ± 4	-1.4 ± 5	-2.4 ± 3.8
**delta End-systolic volume (ml)**	5 ± 3	-4.2 ± 3.1	-8.2 ± 2.4

Data are mean ± s.e.m.

Supplementary Table 10 Serial measurement of left ventricular (LV) function by cMRI of animals treated ICIV and IVIV in a pig model post-MI HF.

	Placebo			Low dose			Medium dose			High dose		
	**Baseline**	**Day 3**	**Day 56**	**Baseline**	**Day 3**	**Day 56**	**Baseline**	**Day 3**	**Day 56**	**Baseline**	**Day 3**	**Day 56**
**LV ejection fraction (%)**	54.1 ± 0.7	37.6 ± 0.3	37 ± 1.1	55 ± 0.8	37.5 ± 0.5	39.3 ± 1.1	53.9 ± 0.7	38.1 ± 0.3	43.6 ± 1.4***	54.7 ± 0.7	36.4 ± 1	44 ± 1.3***
**LV mass (g)**	52.8 ± 2.4	58.3 ± 2.1	62.3 ± 4.3	50.1 ± 2.4	58.4 ± 2.7	68.7 ± 3.9	49 ± 2.3	56.3 ± 2.7	66.1 ± 3.2**	51.1 ± 3.4	56.7 ± 3	62.8 ± 4.3
**End-diastolic volume (ml)**	61.8 ± 3.3	64.9 ± 2.6	77.8 ± 2.8**	62.6 ± 3.4	67.1 ± 3	84.8 ± 3.4***	60.4 ± 3.1	62.3 ± 2.6	73.8 ± 3.7*	62.6 ± 2.6	63.6 ± 2.9	74.3 ± 3.2*
**LVEDV Index (ml/kg)**	2.3 ± 0.1	2.4 ± 0.1	2.7 ± 0.1	2.3 ± 0.1	2.5 ± 0.1	2.6 ± 0.1	2.3 ± 0.1	2.3 ± 0.1	2.4 ± 0.1	2.4 ± 0.1	2.5 ± 0.1	2.5 ± 0.1
**End-systolic volume (ml)**	28.5 ± 1.7	40.5 ± 1.7	49.2 ± 2.1**	28.3 ± 1.7	41.9 ± 1.9	51.7 ± 2.6**	28 ± 1.5	38.5 ± 1.6	41.5 ± 2.1	28.5 ± 1.4	40.5 ± 2	41.9 ± 2.4
**LVESV Index (ml/kg)**	1 ± 0.1	1.5 ± 0.1	1.7 ± 0.1	1 ± 0.1	1.5 ± 0.1	1.6 ± 0.1	1 ± 0.1	1.4 ± 0.1	1.3 ± 0.1	1.1 ± 0.1	1.6 ± 0.1	1.4 ± 0.1
**Stroke volume (ml)**	33.3 ± 1.7	24.4 ± 1	28.6 ± 1.3*	34.4 ± 1.8	25.2 ± 1.2	33 ± 1.3***	32.5 ± 1.7	23.7 ± 1.1	32.4 ± 1.9***	34.1 ± 1.3	23.1 ± 1.2	32.4 ± 1.3***
**Cardiac output (l/min)**	3.4 ± 0.2	2.2 ± 0.1	2.1 ± 0.1	3.3 ± 0.2	2.1 ± 0.1	2.7 ± 0.2**	3 ± 0.2	2.1 ± 0.1	2.6 ± 0.2**	3.3 ± 0.2	2 ± 0.2	2.5 ± 0.2
**Heart Rate (bpm)**	102.9 ± 4.7	91.9 ± 3.9	75.5 ± 3.7**	96.7 ± 4.6	86.7 ± 4.6	85 ± 6.4	94.5 ± 3.9	89 ± 2.8	80.8 ± 2.4	96.9 ± 4	82.4 ± 5	75.8 ± 3.8

Data are mean ± s.e.m.

**P* < 0.05. ***P* < 0.01. ****P* < 0.001 between Day 56 and Day 3 per dosing group; unpaired two-sided Mann-Whitney *U* test.

Supplementary Table 11 Changes in left ventricular (LV) function between day 3 and day 56 (ICIV and IVIV treated animals) in a pig model post-MI HF.

	Placebo	Low dose	Medium dose	High dose
**delta LV ejection fraction (%)**	-0.7 ± 1	1.9 ± 1	5.6 ± 1.2***	7.5 ± 1.1***
**delta End-diastolic volume (ml)**	13 ± 2.8	17.7 ± 3.2	11.6 ± 3	10.7 ± 3.1
**delta End-systolic volume (ml)**	8.7 ± 2.2	9.8 ± 2.4	2.9 ± 2	1.5 ± 2

Data are mean ± s.e.m.

****P* < 0.001; Kruskal-Wallis test with Dunn’s multiple comparison.

Supplementary Table 12 Changes in left ventricular (LV) function between day 3 and day 56 relative to Placebo. (ICIV and IVIV treated animals) in a pig model post-MI HF.

	Relative to Placebo		
	**Low dose**	**Medium dose**	**High dose**
**delta LV ejection fraction (%)**	2.5 ± 1	6.3 ± 1.2	8.2 ± 1
**delta End-diastolic volume (ml)**	4.7 ± 3	-1.4 ± 3	-2.3 ± 2.9
**delta End-systolic volume (ml)**	1.1 ± 2.3	-5.8 ± 2	-7.3 ± 1.9

Data are mean ± s.e.m.
