# Supplementary material for: Preclinical development of a miR-132 inhibitor for heart failure treatment
Source: Nat Commun. 2020 Jan 31;11:633. doi: 10.1038/s41467-020-14349-2 (PMC6994493; doi:10.1038/s41467-020-14349-2)
Supplement: Supplementary file 2 — Reporting Summary [file 41467_2020_14349_MOESM2_ESM.pdf]

nature research

Corresponding author(s): Thomas Thum

Last updated by author(s): Dec 18, 2019

Reporting Summary

Nature Research wishes to improve the reproducibility of the work that we publish. This form provides structure for consistency and transparency in reporting. For further information on Nature Research policies, see Authors & Referees and the Editorial Policy Checklist .

Statistics

For all statistical analyses, confirm that the following items are present in the figure legend, table legend, main text, or Methods section.

n/a	Confirmed	
☐	☒	The exact sample size (*n*) for each experimental group/condition, given as a discrete number and unit of measurement
☐	☒	A statement on whether measurements were taken from distinct samples or whether the same sample was measured repeatedly
☐	☒	The statistical test(s) used AND whether they are one- or two-sided<br>*Only common tests should be described solely by name; describe more complex techniques in the Methods section.*
☒	☐	A description of all covariates tested
☐	☒	A description of any assumptions or corrections, such as tests of normality and adjustment for multiple comparisons
☐	☒	A full description of the statistical parameters including central tendency (e.g. means) or other basic estimates (e.g. regression coefficient) AND variation (e.g. standard deviation) or associated estimates of uncertainty (e.g. confidence intervals)
☐	☒	For null hypothesis testing, the test statistic (e.g. *F*, *t*, *r*) with confidence intervals, effect sizes, degrees of freedom and *P* value noted<br>*Give P values as exact values whenever suitable.*
☒	☐	For Bayesian analysis, information on the choice of priors and Markov chain Monte Carlo settings
☒	☐	For hierarchical and complex designs, identification of the appropriate level for tests and full reporting of outcomes
☐	☒	Estimates of effect sizes (e.g. Cohen's *d*, Pearson's *r*), indicating how they were calculated

Our web collection on statistics for biologists contains articles on many of the points above.

Software and code

Policy information about availability of computer code

Data collection	Echocardiography: Vevo LAB 3.1.0<br>single cell measurements: Axopatch 200B amplifier<br>single cell measurements: IonWizard version 6.5<br>Mass spectrometry: Xcalibur 4.1<br>qRT-PCR: QuantStudio RT-PCR version 1.1. and 1.3
Data analysis	Echocardiography: Vevo LAB 3.1.0<br>Echocardiography: VevoStrain within Vevo LAB 3.1.0<br>Single cell measurements: ClampFit<br>Single cell measurment: JPCalc (see reference, Bary et al., J. Neurosci. Methods 51, 107–16 (1994))<br>Single cell measurements: IonWizard version 6.5<br>Mass spetrcometry: Proteome Discoverer 1.4.0.288<br>Mass spectrometry: Scaffold 4.8.6<br>Mass spectrometry: Mascot 2.3.01<br>cMRI evluation: Segment for Windows version 1.9<br>Volumetric measurements of left and right ventricle: Segment version 1.9<br>Fibrosis calculation: ImageJ version 1.51<br>qRT-PCR: QuantStudio RT-PCR version 1.1. and 1.3<br>Cell size measurement: NIS-Elements BR 3.2 package<br>Data presentation and statistics: GraphPad version 7.0

For manuscripts utilizing custom algorithms or software that are central to the research but not yet described in published literature, software must be made available to editors/reviewers. We strongly encourage code deposition in a community repository (e.g. GitHub). See the Nature Research guidelines for submitting code & software for further information.

Data

Policy information about availability of data

All manuscripts must include a data availability statement. This statement should provide the following information, where applicable:
- Accession codes, unique identifiers, or web links for publicly available datasets
- A list of figures that have associated raw data
- A description of any restrictions on data availability

Figures and Extended Data Figures have associated raw data. There are no publicly available datasets. Raw data for additional information required to interpret, replicate or build upon the findings of this study are available from the corresponding author upon reasonable request.

Field-specific reporting

Please select the one below that is the best fit for your research. If you are not sure, read the appropriate sections before making your selection.

[x] Life sciences   [ ] Behavioural & social sciences   [ ] Ecological, evolutionary & environmental sciences

For a reference copy of the document with all sections, see nature.com/documents/nr-reporting-summary-flat.pdf

Life sciences study design

All studies must disclose on these points even when the disclosure is negative.

Sample size	Sample size for each experiment is indicated in the legend.<br>No statistical tests were used to pre-determine sample size, but sample size was chosen based on previous experiments and comparable studies in literature.
Data exclusions	Based on predefined criteria (Pig study: Animal exclusion due to defined EF criterium for day 3.)
Replication	Not applicable for in vivo experiments. In vitro experiments for screeing (Mass Spec, RNA Sequencing) were performed exploratory with technical replicates, but without biological replication.
Randomization	Animals were randomly assigned to groups.
Blinding	Pig study: Group assignment was blinded during experimental performance and data analysis.

Reporting for specific materials, systems and methods

We require information from authors about some types of materials, experimental systems and methods used in many studies. Here, indicate whether each material, system or method listed is relevant to your study. If you are not sure if a list item applies to your research, read the appropriate section before selecting a response.

Materials & experimental systems

n/a	Involved in the study	
[x]	[ ]	Antibodies
[x]	[ ]	Eukaryotic cell lines
[x]	[ ]	Palaeontology
[ ]	[x]	Animals and other organisms
[x]	[ ]	Human research participants
[x]	[ ]	Clinical data

Methods

n/a	Involved in the study	
[x]	[ ]	ChIP-seq
[x]	[ ]	Flow cytometry
[x]	[ ]	MRI-based neuroimaging

Animals and other organisms

Policy information about studies involving animals; ARRIVE guidelines recommended for reporting animal research

Laboratory animals	Mouse: transgenic mouse overexpressing cardiomyocyte-specific miR-132C57BL/6N, male, 6 weeks;<br>Pig: Mangalica breed, female, 4 months
Wild animals	The study did not involve wild animals.
Field-collected samples	The study did not involve samples collected from the field.
Ethics oversight	All mouse experiments were performed in accordance to relevant regulations and guidelines of the Federation of European Laboratory Animal Science and with approval of the governmental animal ethics committee LAVES (Nds. Landesamt für

Verbraucherschutz und Lebensmittelsicherheit); Animal Welfare Committee of the University of Kaposvar: Animal Study Registration Numbers SOI/31/26-11/2014 and SOI/31/01473-9/2017.

Note that full information on the approval of the study protocol must also be provided in the manuscript.
